# Trabecular bone organoids: a micron-scale ‘humanised’ prototype designed to study the effects of microgravity and degeneration

**DOI:** 10.1038/s41526-021-00146-8

**Published:** 2021-05-21

**Authors:** Alexandra Iordachescu, Erik A. B. Hughes, Stephan Joseph, Eric J. Hill, Liam M. Grover, Anthony D. Metcalfe

**Affiliations:** 1 School of Chemical Engineering, University of Birmingham, Edgbaston, Birmingham UK; 2 Healthcare Technologies Institute, University of Birmingham, Edgbaston, Birmingham UK; 3 The Binding Site, Edgbaston, Birmingham UK; 4 School of Biosciences, College of Health and Life Sciences, Aston University, Birmingham, UK

**Keywords:** Chemical engineering, Osteoporosis, Biological techniques, Tissues, Biomineralization

## Abstract

Bone is a highly responsive organ, which continuously adapts to the environment it is subjected to in order to withstand metabolic demands. These events are difficult to study in this particular tissue in vivo, due to its rigid, mineralised structure and inaccessibility of the cellular component located within. This manuscript presents the development of a micron-scale bone organoid prototype, a concept that can allow the study of bone processes at the cell-tissue interface. The model is constructed with a combination of primary female osteoblastic and osteoclastic cells, seeded onto femoral head micro-trabeculae, where they recapitulate relevant phenotypes and functions. Subsequently, constructs are inserted into a simulated microgravity bioreactor (NASA-Synthecon) to model a pathological state of reduced mechanical stimulation. In these constructs, we detected osteoclastic bone resorption sites, which were different in morphology in the simulated microgravity group compared to static controls. Once encapsulated in human fibrin and exposed to analogue microgravity for 5 days, masses of bone can be observed being lost from the initial structure, allowing to simulate the bone loss process further. Constructs can function as multicellular, organotypic units. Large osteocytic projections and tubular structures develop from the initial construct into the matrix at the millimetre scale. Micron-level fragments from the initial bone structure are detected travelling along these tubules and carried to sites distant from the native structure, where new matrix formation is initiated. We believe this model allows the study of fine-level physiological processes, which can shed light into pathological bone loss and imbalances in bone remodelling.

## Introduction

**Fig. 1:**
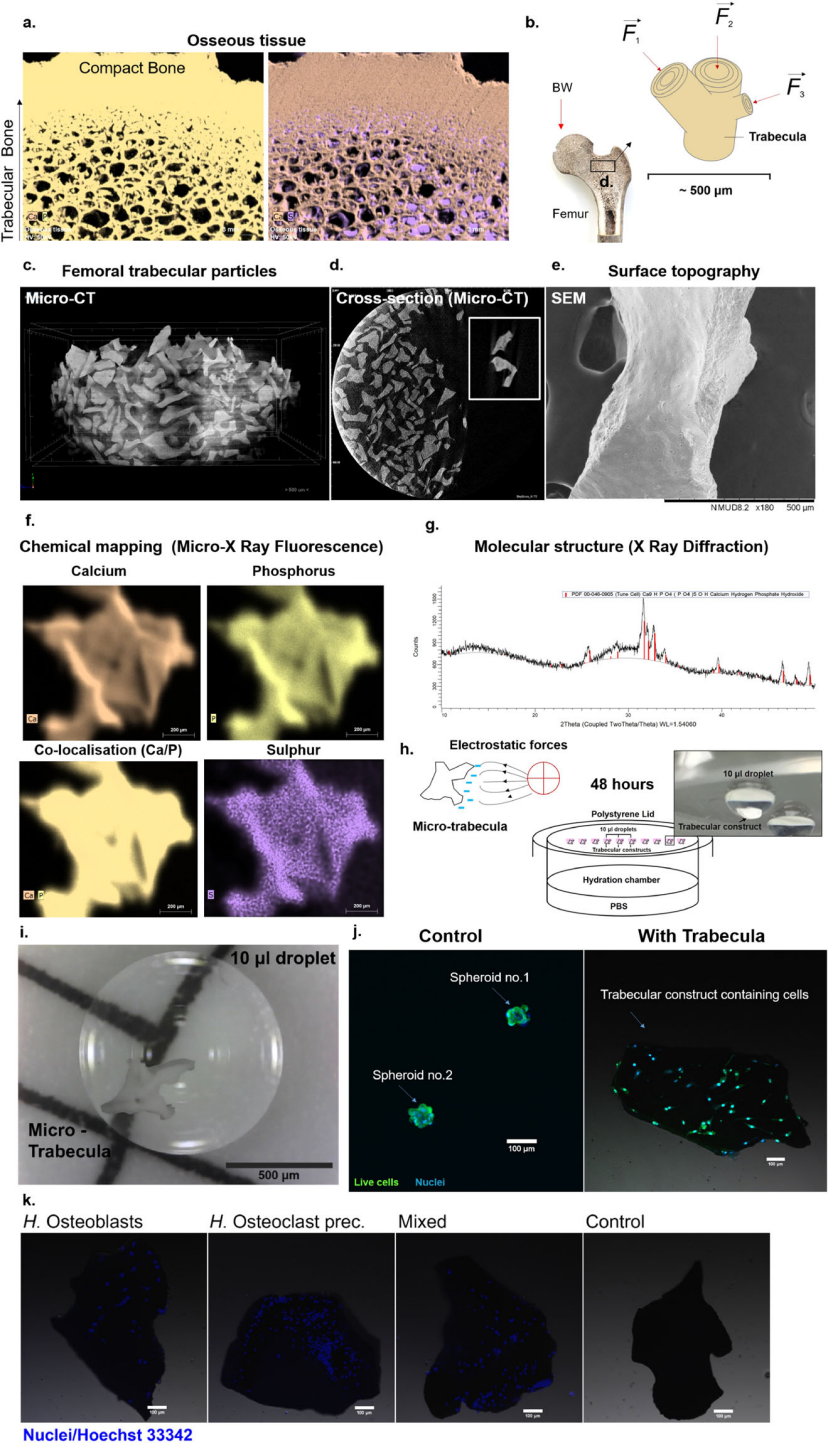
The development of an analogue bone system with equivalent anatomical, biophysical and biochemical characteristics. **a** Bone tissue (exemplified using a *C. elaphus* sp. antler) is composed of a network of trabecular rods, packed together to become compact bone (left). This tissue is heavily mineralised, composed of Calcium Phosphate (left), with little protein content (right), where sulphated content is detected in marrow cavities. **b** The femoral head is adapted to withstand great mechanical loads such as the upper body weight (BW), and is therefore composed of large amounts of trabecular bone (black box). Each trabecula is adapted at the micron level to withstand forces acting from multiple directions. **c** To ensure an anatomically relevant structural base for a bone organoid, thermally treated micro-trabecular particles from femoral heads were used (500–1000 μm). These particles present the lamellar structure (**d**, box) of bone and surface topography (**e**) that is essential for normal cell sensing and attachment. They also have a relevant biochemical composition, comprised entirely of a calcium-phosphate phase (Micro-XRF) (**f**). Traces of sulphur indicative of organic matter can be detected, however, at the limit of the detection threshold. X-ray diffraction analysis (**g**) confirmed these are composed of a biologically derived phase of hydroxyapatite (red bars), the mature bone mineral. **h** The micro-trabeculae are naturally highly electrostatic (left), which was exploited for insertion into liquid cell suspension droplets (right) to generate miniaturised bone avatars. A hanging-drop culture system was used to suspend trabeculae and primary female bone effector cells and to direct attachment onto the trabecular surface through gravitational sedimentation (**h**, **i**). These were cultured for 48 h to maximise surface colonisation and self-organisation of the cells. In the absence of bone scaffolds, osteoblasts come into direct contact with each other to form a spheroid (**j**, left), whereas in the presence of a trabecula they successfully populate the surface and display osteogenic phenotypes (right). Primary female osteoclast precursors are also able to attach individually and co-cultured with osteoblasts, generating a complete remodelling unit (**k**). Their presence can be differentiated on trabeculae as osteoblastic nuclei are larger than osteoclastic ones. Scale bars as indicated.

Bone is an organ that is extremely dynamic and adaptive to its environment, a form of skeletal plasticity shaped by gravity, which has evolutionarily generated changes in all amphibious, terrestrial and volant organisms. Across species, these structural adaptations always manifest in either the densification or reduction of bone content in response to mechanical loads, ranging from pneumatisation of this tissue in volant species to reduce the metabolic costs of powered flight^1^ to osteological densification in land tetrapods invading secondary, such as aquatic niches^2^. In the human skeleton, the development of superior biomechanics allowing verticalisation, including curvatures of the spine, long and hip bone development, meant that the anatomic design was also a variable strategy to increased metabolic and mechanical demands. These adaptations can be seen at multiple scales, most importantly in the micro-structural design as a result of weight distribution. The acting tensile and compressive forces directed that bone was organised at the tissue level in two patterns of arrangement to mechanically support against a wide spectrum of distortive forces. Cortical bone, lining the outside of skeletal structures, manages compressive and tensile forces acting vertically. Within the borders of these cortices, trabecular bone is contained, an essential type of porous tissue mainly present at sites of great mechanical stress, such as the femoral head, where mechanical transfer of weight to the lower limbs takes place (Fig. 1a, b). The trabecular system contains a network of rods, a distribution that allows this organ to withstand forces acting from multiple directions, including bending forces (Fig. 1b). At the micron scale, this is evident as each trabeculae has a lamellar structure that is aligned biochemically with its direction^3^. Because of such thorough mechanical adaptations, this type of bone tissue is also extremely prone to changes caused by reduced mechanical stimulation. In many clinical contexts such as disuse osteoporosis^4^, ageing^5^, spinal cord injury^6^, immobilisation in bedridden/paraplegic patients^7^ and weightlessness experienced by space crews in microgravity^8^, a rapid and significant loss in bone mass in the load-bearing regions takes place (including lower limbs, spine and hip), increasing the risk of fractures^9^ and impairing the healing process^10^. In all contexts, a similar osteopenic to osteoporotic-type gradual loss of mineral takes place, leading to a reduction in trabecular bone density and a porous appearance.

These pathological contexts are difficult to study using current technologies for numerous reasons. Prolonged bed-rest studies in human subjects provide useful physiological data^11,12^, however, most often, the thresholds in experimental design have to be lowered to minimise long-term damage such as alterations in cardiovascular function^13^. In contrast, there are well-established animal models for studying musculoskeletal disuse and bone loss. Rodent models are a frequent choice^14^ and involve either hormonal interventions, including surgical excision of ovaries^15^, hypophysis^16,17^ or parathyroid glands^18^; chemical interventions (diet low in calcium or toxin injection^19^), immobilisation, or a combination of these methods^20,21^, thus removing structures or glands that play a key role in supporting the bone formation process. This is not always desirable, as applying hormonal interventions will likely cause sensorimotor impairment and premature ageing of animals^22,23^. Immobilisation can involve surgical resection of nerves^24^, tendons^25^ or the spinal cord^26^. Some of the more conservative methods involve limb casting or bandaging, but most often use a suspension method, where the tails of animals are attached to the roofs of their habitat cages^27,28^. This facilitates hindlimb unloading for significant periods of time and encourages mobility using the front limbs. One major issue with this technique is that the distress caused by tail-handling^29^ is detrimental not only to the animal used, but could interfere with the secretome being investigated, as fight-or-flight responses are also mediated through hormonal secretion by bone^30^. In addition, the translation of findings to human patient outcomes is difficult, due to differences in anatomical design and weight distribution, in the bone remodelling process between the species^14^ and between strains of the same species^19^. This can be a major bottleneck in the search for promising therapeutics for osteoporosis and related conditions.

Lastly, the bone formation process is extremely difficult to study in vivo and ex vivo due to its highly mineralised nature, which is impenetrable and binds many chromogenic agents and as such requires destruction in order to detect internal histological features. This invasive processing often leads to the destruction of the embedded cellular network, which is vital for understanding the tissue hierarchy and the way cells convert mechanical stimuli into biochemical signals.

At the cellular level, bone formation is temporarily and spatially coupled, relying on the balance between bone effector cells—the tissue building osteoblasts, their mature phenotype (osteocytes), and the non-native bone resorbing osteoclasts derived from monocyte precursors; all under the effect of mechanical stimulation, which is then translated into signalling routes involving hormones and other endocrine organs. Such a microenvironment is therefore very difficult to replicate in a laboratory setting and new ex vivo models recapitulating bone remodelling events are highly needed to overcome these limitations and complement existing systems.

Organoids and organotypic cultures are becoming an increasingly complex platform for replicating anatomical structures similar to human organs, which are unique as they are self-organising and develop features that can be exploited to understand disruptions in molecular pathways that lead to disease. They also have significant advantages for applications in drugs screening and personalised medicine due to their reduced size, allowing for mass applications and numerous permutations of subjected conditions. Over the recent years, various methodologies have been optimised to develop different organoid constructs resembling mainly soft and connective tissues, with most advanced versions being intestinal^31^, including small intestinal^32^ and colonoids^33^; cerebroids^34^, kidney^35^, liver^36^, uterine endometrium^37^ and very recently, hair-bearing skin organoids^38^. However, the repertoire is much wider (see ref*.* ^39^ for a review). In the area of bone, an organoid model is not fully existent, with developmental stages revolving around a spheroid morphology^39,40^. This is likely because of the technical difficulty compared to softer tissues, as the formation of bone constructs requires the careful orchestration by progenitor cells of soft matrix deposition followed by a significant inorganic phase, which has to develop the correct physical alignment at the molecular level, as well as a biologically active mineral phase.

The earliest version dates back to the year 2000^41^ when bone-cell-formed spheroids developed micro-crystalline mineral particles termed spicules; while most recently (2019) spheroids that recapitulate stages of endochondral bone formation were reported^42^, with also preliminary reports of irregular, fibrous bone organoids^43^. In all cases, these model systems focused on a developmental/embryological type evolution, however, no model for a mature phenotype has been developed as of yet, which would be essential for understanding the imbalances in pathological bone formation, which is coordinated by mature osteoblastic/clastic phenotypes. We have, ourselves, recently (2017) achieved to grow a self-organising organotypic bone system using highly osteogenic periosteal cells, which recapitulated the bone deposition process and matured for periods beyond one year of culture, replicating many stages of physiological tissue and apatite mineral phase formation^44–46^. These constructs were designed to be large (at the centimetre scale), as they were scoped for applications in regenerative medicine rather than fine-detail developmental research. Most importantly, however, this was a static model with a longitudinal morphology. In order to add a further level of complexity in the microenvironment, relating to the need of applying mechanical over/under-stimulation as a key culture condition, a smaller scale system was required, that could be inserted into various perfused bioreactor systems currently available. Thus, we have now adapted aspects of our previous technology at the micron scale, to develop a micro-organ prototype of bone that can increase complexity beyond the spheroid stage. We present here the development of this organoid prototype developed solely with primary human bone effector cells, which becomes anatomically relevant and suitable in its properties to be inserted into a free-falling, analogue microgravity reactor for the study of degenerative effects induced by low-shear mechanical stimulation. This manuscript presents results from the three stages of organoid development, from cultivation as a complete bone remodelling unit to encapsulation into a physiological matrix and culture in a mechanically altered environment. We show that the model can function as a complete unit, and that fine-detail early-stage cellular processes can be detected and recapitulated.

## Results and discussion

### The physiological context

Despite their inert appearance, bones are responsible for more than providing a framework for mechanical support. They are continuously remodelled proportionally to the mechanical needs^47^ and in the absence of mechanical stimulation they will shift towards catabolic homeostatic mechanisms to address only the limited amounts of stress they experience. They are also an endocrine organ that fulfils a panoply of vital functions, including providing a source of calcium and phosphate to the rest of the systems and maintaining homeostasis of these elements in the bloodstream under the control of the parathyroid hormone (PTH). New lines of research showed that its matrix also provides a supply of growth factors, including insulin-like growth factors^48,49^, transforming growth factors such as TGF‐β_1_, TGF‐β_2_^50^ and more recently the hormone osteocalcin thought to regulate distant functions such as glucose homeostasis, adiposity^51^ and stress response^30^. Thus, the simultaneous physiological processes undertaken by bone make it very difficult to maintain their health and restore their normal composition. This is because both mechanical loading and the hormone-controlled release of ions to the body continuously require these elements. Therefore, the ionic balance can be quickly disrupted, as seen in numerous pathological states. As such, a system that can provide both conditions would be a step forward in the development process. In this context, the present work focused on developing a method to generate a micron-scale trabecular construct that is biochemically relevant and sufficiently light to be inserted into an analogue microgravity environment, where bone turnover events can be detected while placed under an altered mechanical environment.

### Design considerations

The architecture of trabecular bone (Fig. 1a), especially at sites such as the femoral head (panel b) is optimised to match strength and stiffness to minimal weight. The mature tissue architecture is thought not to be fully pre-determined genetically, but gained also through bone cell activity, which receive mechanical information and influence this adaptation of form^52^. The use of this substrate was therefore essential to initiate a mature trabecular organoid system and to provide the local cues, required for cell recognition and attachment. Micron-scale trabecular particles (500–1000 μm) derived from femoral heads of xenogeneic sources (bovine femurs due to their anatomical similarity to human bone) were chosen as the base of this system to ensure anatomical-region osseous relevance (panel c). These types of particles are thermally treated and designed for human craniofacial, oncological, and other skeletal reconstruction, where, once implanted, they can integrate with the native tissue over time^53^. For this work, these were ideal anatomically, as they presented the lamellar structure (panel d) encountered in trabecular bone, as well as the surface nano-topography (panel e) required for cell detection and native behaviour. These biologically derived micro-trabeculae are composed entirely of bone mineral, made of calcium phosphate (Fig. 1f and Supplementary Fig. 1) and thus essential for simulating the inorganic component of this organ, while allowing a new, organic phase to form. Importantly, unlike other models, this mineral is biological hydroxyapatite (panel g), with a 44.3% crystalline structure and a 55.7% amorphous phase, stabilised into a hexagonal structure, as determined through X-ray diffraction analysis. This high crystallinity is desirable as the rate of hydroxyapatite resorption by osteoclasts is dependent upon this physical property^54^ and as such, it is important for the generation of a relevant bone loss model and resorption rate, which can be similar to the in vivo context. While other synthetic laboratory-manufactured ceramic structures could be trialled, their absorption rate would need to be optimised to ensure they maintain their volumetric stability and do not degrade very rapidly under osteoclastic activity.

From an animal model replacement/reduction perspective, a technical advantage of these substrates is that they are generally sourced from tissue waste from other industries and in addition, minimal trabecular tissue (1–2 cm^3^) is required to generate a significant number of micro-trabeculae (in the order of hundreds).

### Generation of a trabecular organoid prototype using human cells

The micro-trabeculae are hydrophilic and naturally highly electrostatic (Fig. 1h) and as such can be contained within liquid droplets as small as 10 microlitres (panel i), which makes them ideal for insertion into a hanging-drop culture system (panel h), commonly used as an efficient, cost-effective method for generating spheroids^55^. An important feature of this technique is that, when used with multiple cell phenotypes, these can self-organise into cortical and central configurations, in a surface-tension directed manner^56^. These cellular kinetics were exploited in this work for directing attachment of human bone cells, in individual and mixed forms. These cells aggregate, under the action of gravity, on the surface of the micro-trabeculae after being allowed to sediment for a period of 48 h to maximise self-organisation, attachment and cell-cell contact. Bone forming human osteoblasts, when cultured using the traditional method are able to form spheroids (panel j, left), whereas when the micro-trabecular component is added, they are able to colonise the surface and express more relevant phenotypes (panel j, right). The system allows the attachment of both human osteoblasts and osteoclasts, the latter as either mature and pre-osteoclastic monocytes; as well as a combination of the two cell types (panel k). Examples from each category are shown in Fig. 1k following 48 h inside their respective droplets.

### Cytodifferentiation in organoids and mimicry of tissue functions

Bone remodelling takes place under the action of a carefully orchestrated and synchronised feedback system, involving three types of bone effector cells, originating from different lineages: the mesenchymal-derived, matrix secreting osteoblasts, their mature phenotype (osteocytes) involved in mechanosensing and maintenance^57^, and non-native pre-osteoclasts originating from hematopoietic lines, which migrate as monocytes to the site as required, where they differentiate near or at the bone surface and undertake a highly destructive bone resorption activity^58^. The balance between resorption-deposition is disrupted in osteopenia/osteoporosis and it has become clear over the recent years that it is not the isolated effect of one cell type, but the altered response of the entire unit, which leads to a reduction in bone mass^59^. This became very evident particularly following space exploration missions^60^, where severe losses of bone mass were observed at an accelerated rate compared to osteoporotic patients, and while osteoblasts showed diminished activity^61^, osteoclastogenesis was significantly increased^62^. Similarly, using the hindlimb immobilisation methods in rodents discussed above, a rapid increase in bone resorption was detected as early as 3 days^63^, while some studies reported a surge in osteoclast numbers per millimetre of bone surface as early as 30 h^64^. On a technical level, these rapid events highlight the need for a model that can capture the early cellular events in such a narrow time window.

Therefore, to understand imbalances in the signalling and communication activity in an in vitro/ex vivo model, these cells ought to be used together in a system, which can ultimately be subjected to altered mechanical forces. Optimisation was performed using individual and mixed-cell constructs and an osteoblast-osteoclast system represented the final set-up to generate a complete remodelling unit that could recapitulate the cell-cell and cell-surface-cell interactions without direct contact of the two populations as with other juxtacrine models. This compatibility has been previously verified by vast amounts of research using aggregates of osteoblasts and osteoclasts, as lumps or separated by a physical barrier on macroscopic matrices (see refs*.* ^65^ and ^66^ for extensive reviews).

At this level, there were further design considerations such as the ratio of osteoblasts to osteoclasts in our mixed model, due to the significantly larger size of the latter, as well as due to number of cells that could colonise the surface, calculated based on previous models^34^, as well as the in vitro surface correspondence. To simulate the bone multicellular unit, we chose this ratio as 1:2–3.5 osteoclast:osteoblast, which fits with previous compatibility ratios reported as most successful for this combination of cell types^67,68^. We have therefore used mixed culture media at a ratio directly proportional to that of cells used, to deliver a controlled mixture of essential growth and differentiation factors to these constructs, including pro-osteoblastic, osteoclastic and osteocytic. Supplementary Fig. 2 provides a detailed summary of the experimental set-up, the supplementation regime and predicted cellular differentiation in every cell category. The ratio of osteoblast to osteoclasts could be adjusted in future studies to reflect different stages of the normal or clinical context.

### Simulation of osteoclastic function

**Fig. 2:**
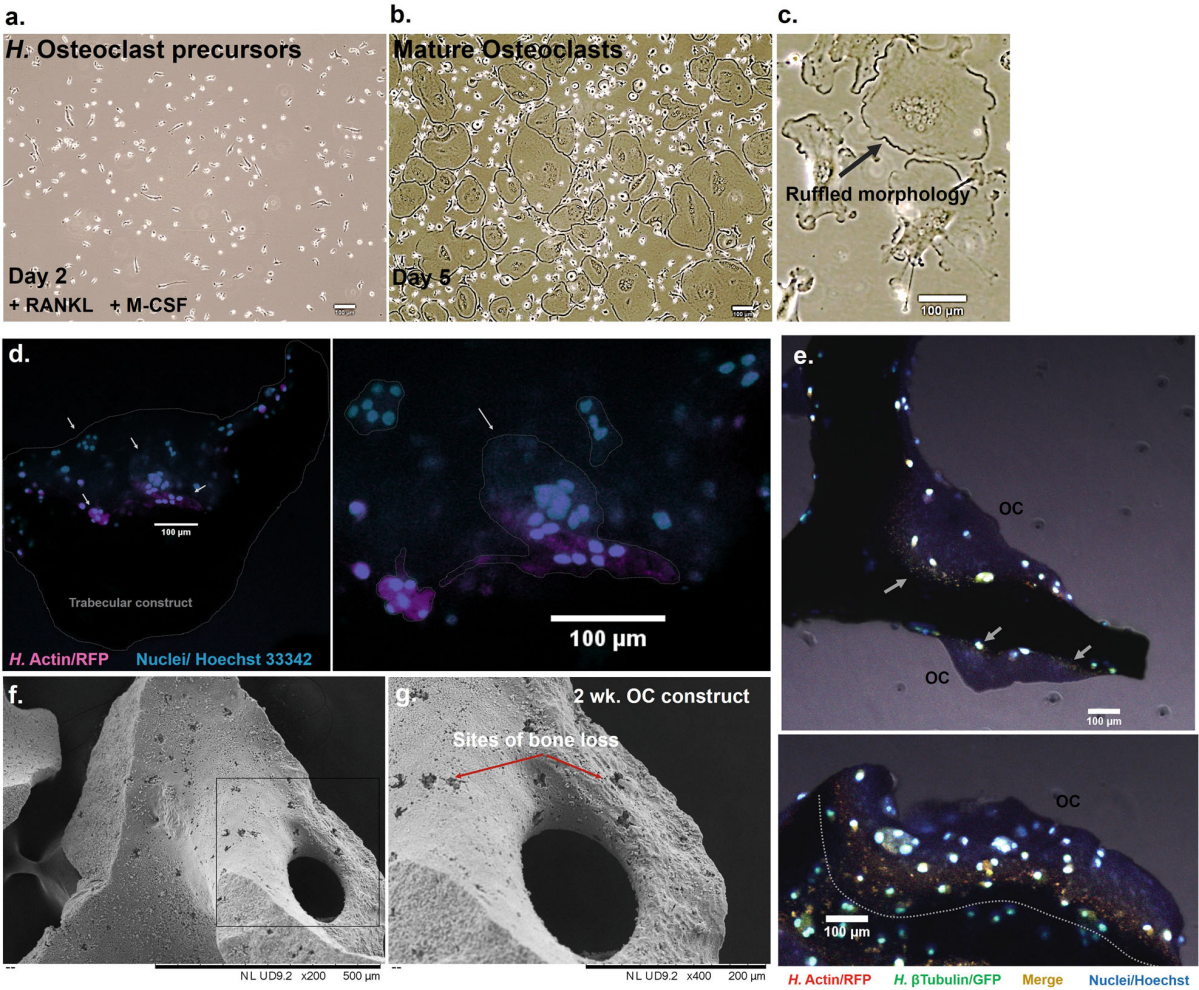
Maturation of the human osteoclastic constructs and simulation of the specialised bone resorption activity encountered in vivo. Human osteoclast precursor cells of monocytic origin (**a**) are incubated in the presence of pro-osteoclastic factors RANKL and M-CSF to generate a population of multi-nucleated osteoclasts, which display the ruffled morphology indicative of their active phenotype (**c**, black arrow). The fusion of monocytes into polykaryons can be observed in constructs as well after 8 days of culture (**d** left and zoomed, right), where they are seen agglomerating on the surface of trabeculae. Some of the formed structures are delimited (dotted lines, arrows) to allow a better visualisation. At the interface with the scaffold, these cell aggregates form belts (**e**, top and bottom) that are rich in tubulin and actin (white arrows and dotted line). When cultured for 2 weeks, these cells can create perforative resorption patterns resembling pores and lacunae (**f**, black box and **g**, red arrows). Scale bars as indicated.

As mentioned above, osteoclasts play a key role in pathological bone loss, which structurally manifests an excessive form of their normal physiological function. Female patient-derived, primary monocyte precursors (Fig. 2a), which had undergone tartrate-resistant acid phosphatase (TRAP) and osteolysis validation as a population, were cultured and differentiated through conditioning with the receptor activator of NF-κB ligand/RANK Ligand (RANKL/TNFSF11), as well as the macrophage-colony stimulating factor (M-CSF). These paracrine factors native to osteoblasts and osteocytes are both necessary and sufficient for osteoclastogenesis and can stimulate immunological precursors in our system to maximally differentiate in a time frame covering up to 5 days (panel b). Following maximal attachment (approximately day 2), these cells merge into very large, multi-nucleated structures, are mobile and contain a typical ruffled border (panel c). The initial organoid development was originally conducted with these matured osteoclasts for concept proofing, which was then simplified for use with osteoclastic progenitors, for both technical and design advantages, as they were easier to isolate, quantify and to simulate the migration and differentiation process at the site during the inversed drop stages. When applied to the micro-trabeculae as precursors, these cells mature into polykaryons and agglomerate into large structures (panel d, left and right). In terms of activity, at the interface with the micro-trabeculae, following 8 days of total culture, we were able to detect contact sites containing a co-localisation of the cytoskeletal proteins actin and β-tubulin (panel e, top and bottom). This is important as sites of cortical actin and tubulin localisation are part of essential podosome belt-like structures in osteoclasts, which are critical for resorption and sealing the attachment point in order for H^−^ and Cl^−^ ions to be transported through the ruffled membrane for dissolving the mineral phase^69^. The ability to detect such fine details in the system displays great promise for applications in understanding more native behaviours of osteoclasts and we have already started to look at the spatial localisation and chemical nature of chlorine on the surface, which so far appears to be independent of a common sodium salt-type association.

When constructs were cultured for 2 weeks in a test experiment, we were able to observe perforative resorptive sites on the trabeculae (Fig. 2f, g), which were the subject of investigation of additional work (Figs. 4 and 5).

**Fig. 3:**
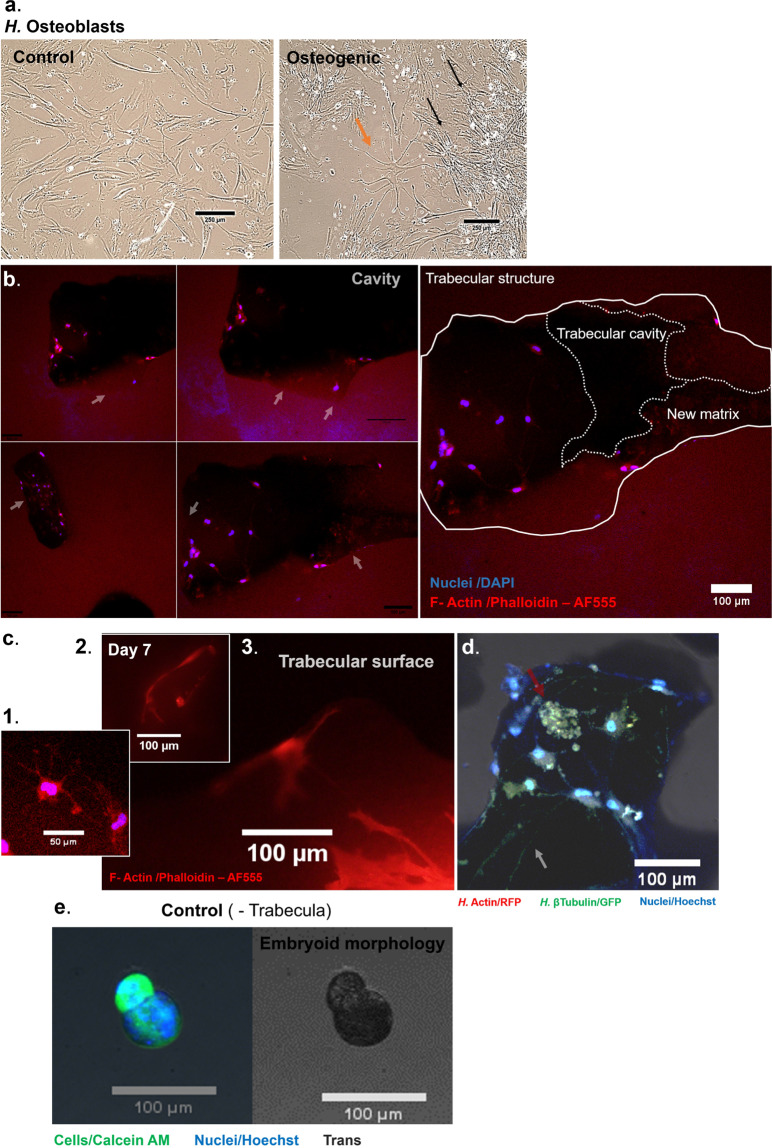
Maturation of the human osteoblastic constructs and simulation of the osteoid producing events encountered in vivo. Human osteoblastic cells cultured in non-osteogenic (control) medium for 14 days (**a**) display an unchanged osteoblastic phenotype, whereas cells cultured in osteogenic medium for this period display a highly dendritic phenotype within an interconnected network, indicative of an osteocytic phenotype (**b**). Constructs seeded with cells and cultured on non-adherent surfaces for 7 days are able to recapitulate in vivo events such as osteoid production. New matrix is produced, which can be morphologically distinguished from the compact bone structure (**b**, left, white arrows). Cavities within trabeculae are used as a sites where new matrix formation takes place (**b**, right), and dendritic cells are seen embedded within this matrix (**c**, 1). The surface of constructs is also a site of osteoblastic-osteocytic activity, with large projections extending across the trabeculae (**c**, 2, 3). In addition, communication and aggregates (red arrow) can be observed at the surface, which are rich in tubulin and actin (**d**). **e** In contrast, constructs that were cultured in the absence of trabecula, including 7 days inside a perfused rotating vessel, develop a lobular embryoid-type morphology. Scale bars as indicated.

**Fig. 4:**
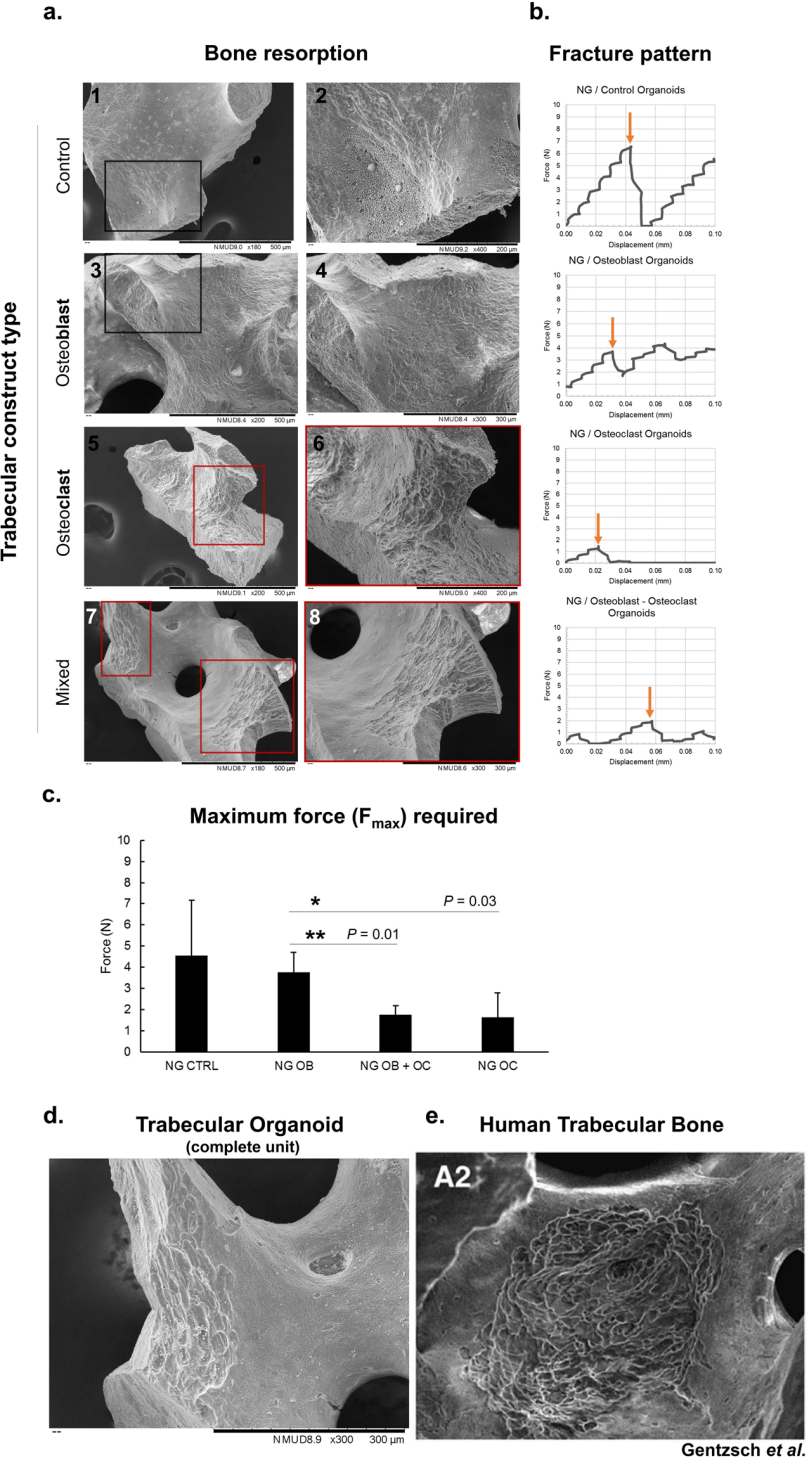
Simulating bone loss in constructs and the effects on local mechanics. Resorption of the bone surface was investigated on trabeculae from different cell-seeded groups after 8 days of culture to detect the manifestations in each type of condition. Cell-free constructs and those that were cultured with osteoblasts show only signs of endogenous resorption, originating from the native bone tissue (**a**-1,2 and 3,4, respectively). Osteoclast-cultured constructs show a significant pattern of resorption (**a**-5, 6), which was observed in mixed constructs as well (**a**-7, 8), developed with both osteoblastic and osteoclastic cells. Dark boxes = areas of no resorption; red boxes = areas of resorption. The mechanical properties of these groups were investigated by subjecting the cultured constructs to compressive forces and observing the fracture patterns, related to their trabecular distribution (**b**), and changes in maximum force required to collapse the structure, a feature related to their integrity (**c**). The values selected for comparison, for which representative examples are presented in **b** with orange arrows, were chosen as the maximum compressive force that can generate the ultimate structural failure. The least-resistant organoids were those that contained mixed osteoblasts-osteoclasts and osteoclasts alone (**p = 0.01 and *p = 0.03, respectively, *n* = 3), which required significantly less force to reach terminal failure. Data is presented as mean ± SD. The resorptive patterns encountered in mixed constructs simulating a whole remodelling unit (**d**) were reticulate in appearance, similar to those encountered in human trabecular bone from femoral heads. Figure **e** is taken from Gentzsch et al.^75^ by permission from Springer Nature. Scale bars as indicated.

**Fig. 5:**
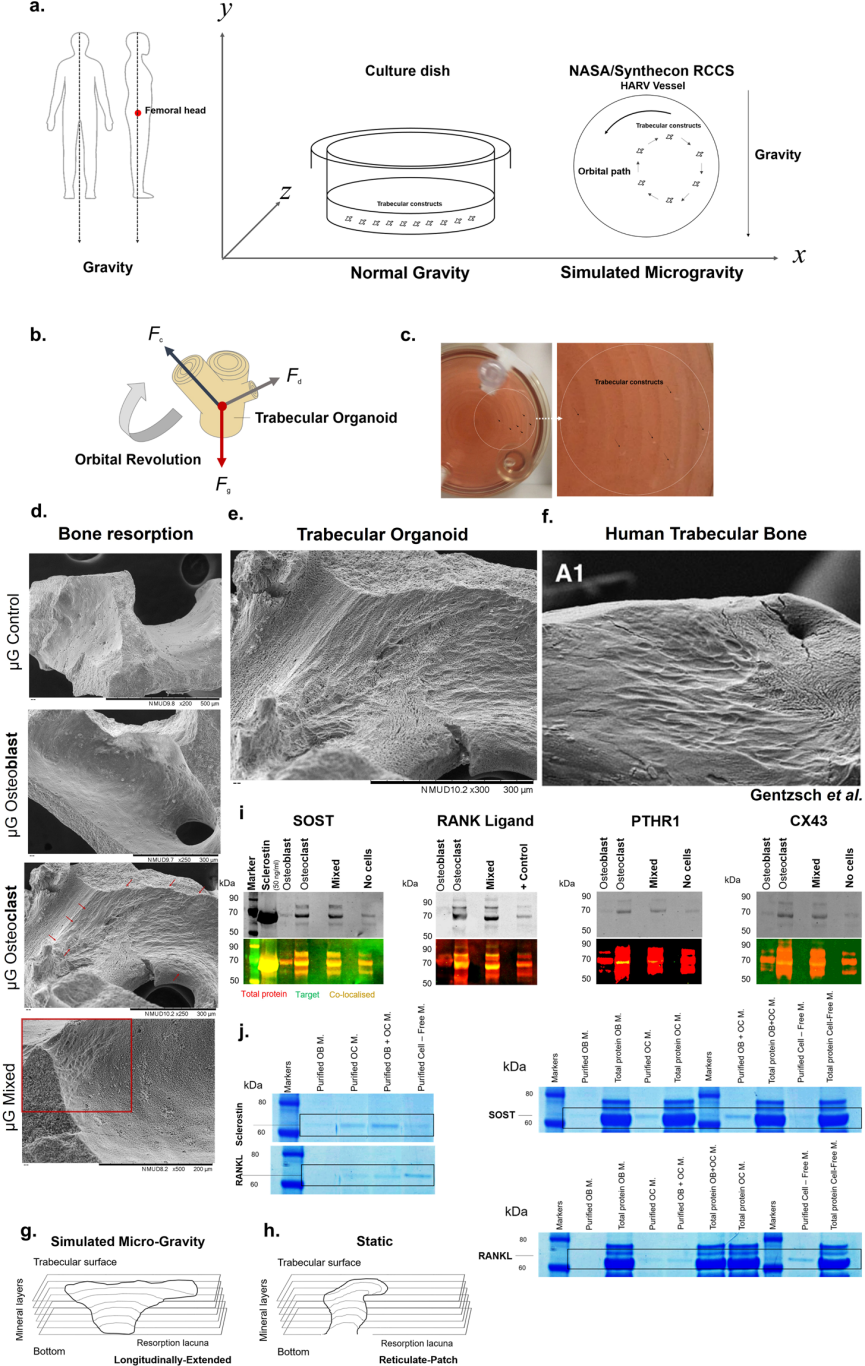
Changes in cell resorption observed with exposure to simulated microgravity. **a** To simulate a reduction in the force of gravity that the femoral head trabeculae are normally adapted to resist, particularly when upright (left), organoids were placed either in static, typical culture conditions, where they experience the normal gravitational pull, or in dynamic, rotational conditions, inside a NASA-synthecon reactor vessel, where a laminar movement of the culture medium suspends constructs in a state of orbital buoyancy (right). During this culture model, the forces acting on the trabecular organoids, resulting from the gravitational force (*F*_g_), centrifugal forces (*F*_**c**_) and hydrodynamic drag forces (*F*_**d**_) (**b**) balance each other during each orbital revolution (**c**), thus preventing sedimentation and simulating weightlessness. Following 6 days of culture under these conditions, resorption induced by cells can be observed in osteoclastic and osteoblastic-osteoclastic constructs (**d**, red arrows and box), which appears longitudinally extended in morphology (**e**). This resorption pattern resembles a second class of lacunae encountered in trabecular bone from human femoral heads (**f**) and is different structurally from the type encountered in constructs cultured under static conditions (**g**, **h**). Extracts from the incubation medium of constructs subjected to simulated microgravity were taken as pellets (**i**), or immuno-purified from liquid phase (**j**). In both cases, large protein complexes were detected between 60 and 70 kDa, containing an association of the target protein, native cell and medium proteins (**j** right, black rectangles). These regions were probed with antibodies during western blotting (**i**), or the protein was immuno-selected using magnetic isolation (**j**, left). The protein Sclerostin (**i**) was detected in osteoblastic samples, but additionally in osteoclast and mixed samples/medium. This pattern was also observed in concentrated medium samples (**j**, left—immuno-selected samples and right—all samples including medium). RANKL (**i**) was not expressed in osteoblast constructs, in the absence of osteoclasts. RANKL was also detected in concentrated medium samples, in all but osteoblast samples (**j**). The endocrine protein PTHR1 and integrin CX43 (**i**) were only detected in osteoclastic constructs. Figure **f** is taken from Gentzsch et al.^75^ by permission from Springer Nature. Scale bars as indicated.

### Simulation of (concomitant) osteoblast function and matrix initiation

**Fig. 6:**
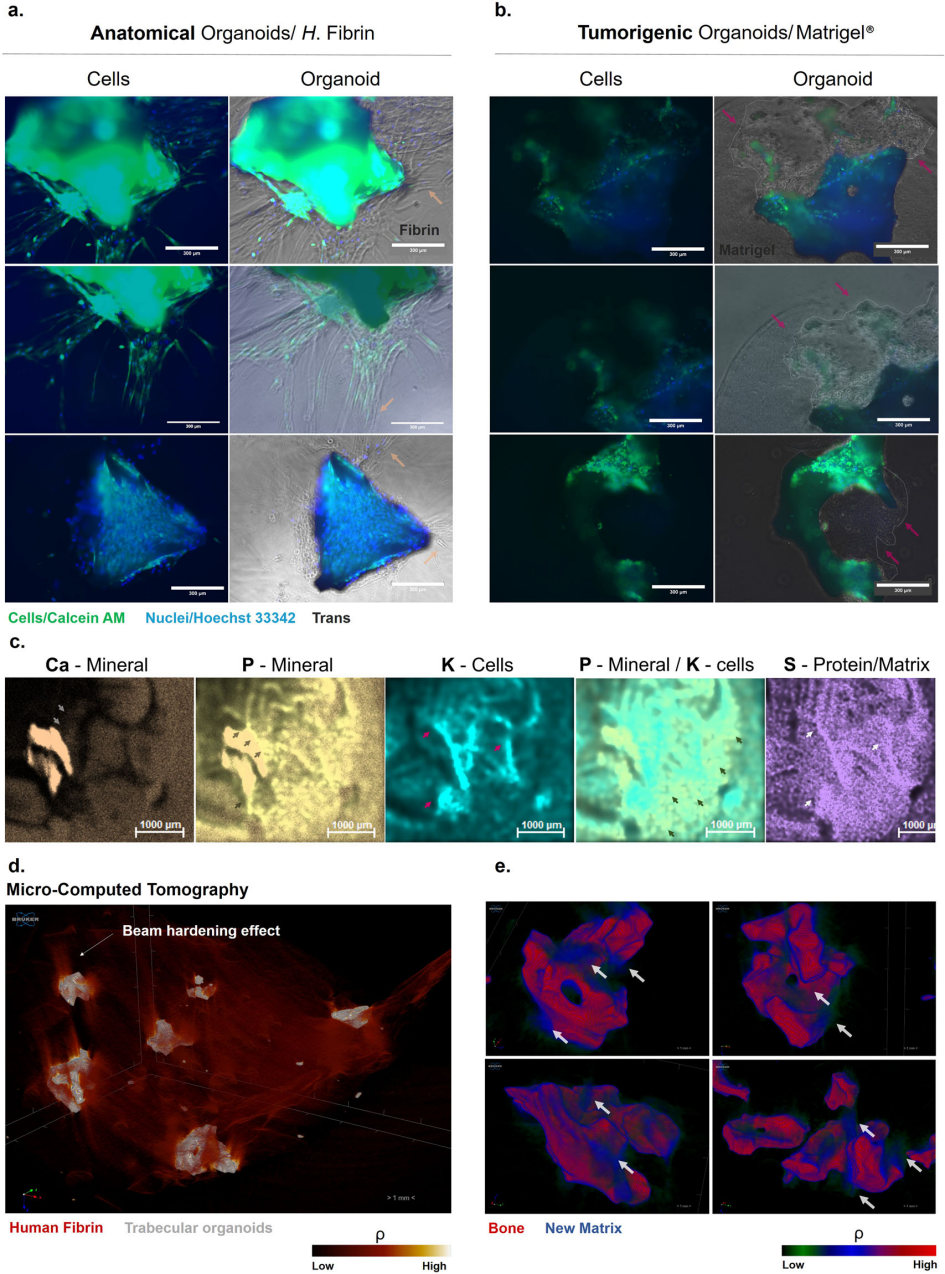
Encapsulation of trabecular organoids into biochemical microenvironments that can generate bone physiological or pathological prototypes. **a** and **b** present two examples each of constructs that were encapsulated into a physiological (human fibrin) and tumorigenic environment (Matrigel^®^). Constructs that were encapsulated in fibrin (day 19) create extensive projections (orange arrows) into the surrounding environment (**a**-top), which stretch from multiple points on the surface (middle). These constructs are heavily populated with cells (bottom). Constructs embedded in Matrigel^®^ (day 16) also display highly proliferative cells, however, the pattern and morphology of these cells is more representative of a tumouroid, as they are densely packed into atypical phenotypes (pink arrows). These lobular proliferative regions have been delimited with a dotted white line for easier visualisation. **c** The chemical composition of fibrin-embedded constructs and nature of projections was probed using X-ray fluorescence mapping to allow for spatial detection of new features, with different elements coding for distinct anatomical structures. The projections emerging from the construct surface (grey arrows and black arrows) contain a large amount of Phosphorus (P). Calcium (Ca) is also present in the newly forming structures. Potassium (K), an intracellular ion present in cells is seen within these dendritic structures (pink arrows) and co-localised with the Phosphorus deposits, however, discrete deposits of P are found on their own surrounding the K structures (green arrows). Areas rich in Sulphur (S) are also seen localised to the regions where projections and matrix are forming (white arrows). Organoids were scanned with Micro-CT as a fused mass (**d**) to detect compositional gradients based on density. Owing to the dense nature of the trabecula, organoids behave like bone tissue when subjected to X-Ray radiation and as such, display selective attenuation at the surface (the beam hardening effect). Therefore, beam artefacts were removed and a function was applied to remove the lower density ranges of data (**e**). White arrows show discrete pockets of a denser (blue) material in different constructs in the near vicinity or emerging from the very dense (red) surface, and trapped within the lowest density (green), surrounding fibrin material. Scale bars as indicated.

**Fig. 7:**
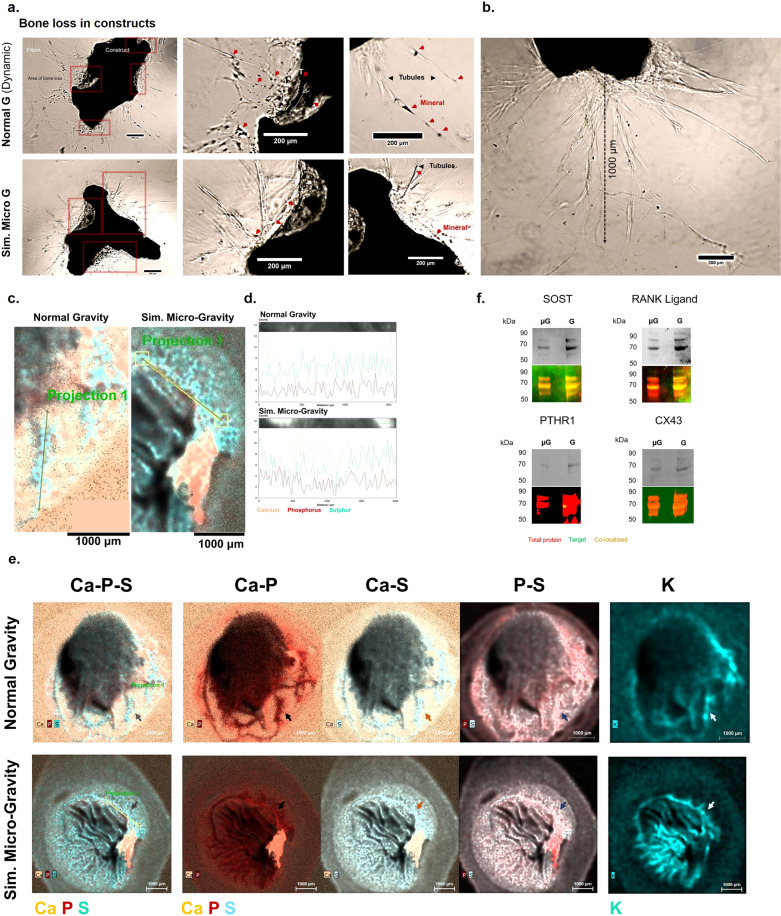
From bone loss to bone remodelling and fracture repair. Mixed-cell organoids were investigated following 23 days of total culture, including 5 days inside a simulated microgravity vessel under two rotating conditions (simulated Micro G and Normal G Dynamic). **a** When imaged using brightfield microscopy, areas of significant bone loss (red squares) can be observed at various locations adjacent to the organoid surface in both conditions. Large tubular projections (black arrows) can be observed emerging in both conditions from the original organoid and into the surrounding fibrin matrix. Some dense mineral-tubular structures (T) are seen forming from the surface (top panel, white dotted lines). These projections carry mineral (red arrows) to sites away from the main structure. These structures can reach ~1 millimetre, as observed in this simulated microgravity-cultured construct (**b**). These large projections were chemically mapped at high resolution using Micro-X-Ray Fluorescence and were shown to contain a mixture of protein and mineral content (**c**, **d**), where the elements Sulphur, Phosphorus and Calcium were co-localised in both conditions along the length of these tubule formations (**d**). **e** Whole maps of constructs revealed intricate networks of mineralised matrix projections also containing cells in both conditions (coloured arrows) and differences could be assessed morphologically. In these particular examples selected, a construct from the normal gravity condition (top) contains a higher number of projections compared to the simulated microgravity construct (bottom), which contains a main tubular structure forming into the matrix, but which displays many potentially emerging structures, based on the distribution of potassium (K), in networks surrounding the initial structure. Molecularly, a non-quantitative protein assessment of constructs revealed that the Sclerostin and RANKL presence was pronounced within each condition compared to that of the endocrine Parathyroid Hormone Receptor 1 and to the shear-responsive Connexin 43 channels (**f**). The presence of PTHR1 in the modelled microgravity group was not clearly detected, however, it was present in the normal gravity condition, which may indicate differences in the regulatory pathway controlling the bone remodelling process between the two contexts.

Female patient-derived osteoblasts were cultured in basal medium supplemented with steroidal and pro-mineralisation supplements (β-Glycerophosphate) to induce more native specialisations. When cultured for 14 days in control medium, these cells maintain a mesenchymal phenotype, whereas with osteogenic supplementation, they maximally transition to a highly dendritic morphology, and are also densely interconnected, suggesting a pre-osteocytic phenotype (Fig. 3a). Further morphologies indicating the ability to form mature osteocytes are also demonstrated in Supplementary Fig. 3. When cultured on micro-trabecula, they are able to develop identical dendritic connections (panel b, c1–3), and some highly stellate looking cells are seen embedded in new matrix formed by cells inside the cavities of the trabeculae (panel b, c1), which are structures of similar architecture and porosity as found in bone. At the surface of constructs, the projections can become extensive following 7 days of culture and contain the cytoskeletal protein β-tubulin, as well as actin (panel d). From an anatomical perspective, these structures are essential, as osteocytic dendrites are key mechanosmes^57,70^, with absence resulting in resistance to unloading bone loss^71^. Secondly, these projections are the likely precursor of greater, more evolved structures discussed later in Figs. 6 and 7. Lastly, the achievement of several phenotypes is desirable for simulating a complete remodelling unit, and the morphologies of cells from all variations, including mixed phenotypes is presented in Supplementary Fig. 4. For comparison, constructs that were formed without micro-trabeculae using the inversed drop and subsequently cultured for 7 days inside a rotary bioreactor in preparation for the final stage, develop from a spheroid (Fig. 1j) into a lobular embryoid-type morphology (Fig. 3e). While interesting from a developmental perspective, the cellular behaviour detected in constructs suggests the need to develop bone organoids of greater complexity than osteospheres.

### Anatomical and micro-mechanical properties of trabecular organoids

Excessive bone resorption in pathological states leads to a reduction in mineral content, which ultimately has a key effect on bone micromechanics and resistance to breakage, significantly increasing the risk of fractures^72,73^ and delaying healing^74^. In order to investigate the degree of mineral resorption led by osteoclasts in this system, organoids were developed with individual and mixed populations of cells as described above. The surface of micro-trabecula was imaged at high-resolution to detect the results of cell activity following 8 days of total static culture (including 6 days in non-adherent conditions). While native endogenous resorption could be observed, as expected, in control and osteoblast trabeculae (Fig. 4a1, 2 and 3, 4, respectively) significant resorption lacunae were identified in osteoclast-containing constructs (panel a5, 6), including the mixed osteoclastic constructs (panel a7, 8). We subsequently trialled a method to investigate whether this loss of tissue was translated into their mechanical resistance characteristics by subjecting organoids to compressive testing. The maximum force required to break these constructs was used as a measure of their mechanical capabilities and peak forces were selected based on their fracture pattern, in some cases characterised by multiple peaks due to the non-geometrical shape of constructs. The maximum force was chosen as the maximal point at which the significant collapse of the structure was achieved. Examples from each category are illustrated in Fig. 4b. Results from this pilot study (panel b, c) revealed that osteoclast resorption leads to a diminishment in mechanical resistance (panel c), as osteoclast-containing organoids required significantly less force to be terminally destructed compared to similar constructs containing osteoblasts (up to 2.91 N in osteoclast, 2.11 N in mixed constructs, compared to 4.86 N in osteoblast constructs). This suggested that it was possible to recreate pathological biomechanics to a basic degree through the addition of human osteoclasts. At a structural level, interesting morphological features were detected (panel d), with the type of resorption lacunae at the surface characterised by a reticulate patch-type, being one of two major types that have also been encountered in trabeculae from human femoral heads (panel e)^75^.

### Subjecting organoids to mechanical unloading: turning a problem on its side

In order to simulate a reduction in the gravitational force that the trabeculae of the femoral head are normally adapted to counteract during an upright position or bipedal ambulation (Fig. 5a), the engineered organoids were inserted into a NASA-Synthecon bioreactor to be subjected to a reduced mechanical loading state following the initial drop culture. This microenvironment was achievable due to their reduced size and weight reported to that of the large (50 ml), high-aspect-ratio rotating vessels (HARVs) of the reactor. During a 360 degrees rotation, the culture medium inside the vessels has a laminar flow, moving as a solid mass^76^, thus creating minimal shear stress on the organoids, which assume a circular trajectory while falling vertically. During rotation of the gravitational vector in this model system, the forces acting on the trabecular organoids (panel b), resulting from the gravitational force (*F*_g_), centrifugal forces (*F*_c_) and hydrodynamic resistance/drag forces (*F*_d_) balance each other, thus preventing sedimentation (panel c) and simulating weightlessness and mechanical unloading. The constructs and their culture media were analysed following 6 days of culture, at a speed of 29 RPM under these buoyancy conditions in order to try and identify any individual or cumulative cell responses at the structural (panel d–h) and molecular levels (panel i, j).

When the surface of organoids was analysed for osteoclastic activity, a similar rate of resorption as seen in static conditions was observed (Fig. 5d), with solely osteoclast and mixed osteoclast constructs displaying new surface resorption. Interestingly, however, the morphological appearance of these resorption pits was significantly different compared to static controls (panel d, e), with only longitudinally extended types of resorption lacunae identified in these organoids, suggesting a difference in cellular kinetics between the two conditions. This type is also the second major style of resorption lacuna identified in trabecula of human femoral heads (panel f)^75^ and a schematic comparison between the two conditions is presented in g. There is an extensive body of work that has tried to provide correlations of these resorptions with taxonomy, age, gender and trabecular positioning with sometimes inconclusive results^75,77^. Therefore, the ability to detect such differences in resorption patterns might constitute a promising way of utilising this model, in a larger scale study.

It was then evaluated whether any differences could be detected in constructs in the cell response when subjected to analogue microgravity. In a pilot study, the differential expression of factors indicative of bone remodelling (Sclerostin, RANKL), endocrine activity (Parathyroid hormone receptor 1) and mechanical sensing (Connexin 43) across groups was investigated (Fig 5i, j). This was performed on extracts from cell membranes that were pelleted through centrifugation (panel i) or immuno-isolated from the liquid phase (panel j) of the mass of culture medium in which the constructs were suspended, to obtain a quick picture of key secretome and receptor activities.

To break down some of these proteins from complexes with other integral membrane proteins that are commonly found in cellular composition, gel electrophoresis was conducted with or without antibody recognition using western Blotting to detect their presence either as monomers, dimers (as expected in biological form) or associated with larger proteins.

Using both techniques, large protein complexes were detected in all cases within the molecular range of 60–70 kDa, which contain an association of the target protein, native cell and medium proteins (Fig. 5j, right black rectangles). When these regions were probed with antibodies during western blotting (panel i), or the protein was immuno-selected from the total sample using magnetic immuno-isolation (panel j) several differences could be detected across the different cell groups.

Sclerostin (SOST), a Wnt/β-catenin signalling antagonist involved in both osteocyte and osteoclast function, and which appeared as a dimer when ran in pure form (Fig. 5i, first panel), was detected in osteoblastic samples, but also interestingly, in osteoclast and mixed osteoblastic-osteoclastic samples. The detection of Sclerostin in osteoblastic constructs suggests that, as noticed morphologically as well, some of the cells have matured into an osteocytic phenotype, as this protein is a signature molecule for the terminally differentiated bone cells^78,79^. Its presence in osteoclastic samples is also interesting. While this protein is also likely present in the osteoclastic culture medium (as indicated by the mixed control medium, which contains this component), as it is supplemented with a cocktail of native osteoblastic additive factors, further, pronounced bands can be detected in the osteoclast-containing groups, suggesting a different expression pattern of this protein in these samples. This was also supported by the immuno-purified samples, which demonstrated a similar expression pattern across samples (panel j, left and right panels). This is of particular importance as Sclerostin is thought to be an inhibitor of bone formation through osteocytic suppression of osteoblastic function, however, similarly to this study, more recent research has shown that osteoclasts of aging mice express Sclerostin themselves, which reduces mineralisation through that direct route^80^. It had also been previously localised in osteoclasts in both intramembranous and endochondral bones^81^ and it was recently shown that osteoclasts can downregulate Sclerostin expression in osteocytes in trabecular bone^82^. These results further highlight the need for a model that can capture these temporal events, due to the multitude of stages involved in the signalling process between osteoblast-clast-cytes.

While bone mass is negatively regulated through an anti-anabolic effect of osteocytes on osteoblasts via Sclerostin secretion, catabolic actions of osteocytes can take place, via recruitment of osteoclasts through secretion and up-regulation of the TNF-related cytokine RANKL, which activates the RANK receptor on the surface of hematopoietic precursor cells^58,83^. Recent studies also suggested that osteocytes, not osteoblasts or lining cells are the major source of this protein^84^. RANKL (Fig. 5i, second panel), was a necessary exogenous factor in this system in order to control the timing of osteoclast maturation. When the levels were compared across different groups, we discovered that this protein was not expressed in osteoblast constructs in the absence of osteoclasts. Mixed and osteoclast constructs do contain higher levels and further bands of this protein compared to controls. RANKL was also detected in concentrated medium samples, where it is similarly present in all but osteoblast samples (panel j, left and right). As the osteoblast constructs contain some of the initial osteoblastic population as well as many transitioning osteocytes, as indicated by the presence of sclerostin and their dendritic phenotype, these findings suggest that the presence of osteoclasts or their precursors in the culture is necessary to induce the expression of this factor, which is also supported by the fact that direct cell-to-cell contact with the RANKL expressing cells is known to be required for osteoclast activation^85^. Therefore, the interplay between the sclerostin-mediated bone inhibition and RANKL expression is something that could be investigated in future studies using this system.

Calcium homeostasis and bone remodelling are also controlled through the action of the Parathyroid hormone (PTH), which has both anabolic and catabolic roles on the cells and bone tissue, stimulating both bone formation and resorption^86^. Interestingly, the receptor involved in this signalling cascade and which has an endocrine role, the Parathyroid Hormone 1 Receptor (PTHR1) (Fig. 5i, third panel), is only expressed in osteoclast-containing constructs. This is interesting firstly because the PTH-mediated homeostasis route is known to be an inhibitor of sclerostin expression^87^ and therefore, the absence of this receptor in osteoblast constructs appears to match the presence of sclerostin. Secondly, because the presence of this receptor in osteoclasts has only become evident recently^88^, first in the bone tissue of deer antlers (Fig. 1a), which similarly to trabecular bone, has a high turnover/remodelling rate. There, the PTH related peptide (PTHrP) is involved in osteoclastogenesis in a RANKL-independent pathway, which suggested that osteoclasts expressing the receptor can be directly stimulated independently of RANKL.

The gap junction protein Connexin 43 (CX43), was detected only in osteoclastic samples, but not in osteoblastic-osteocytic constructs (Fig. 5i, fourth panel). This is interesting because the hemichannels that this protein forms, especially on dendrites, only open with mechanical stimulation in response to fluid flow shear stress^89,90^, which is greatly reduced in this system. This suggests that the anabolic effects of mechanical loading through the CX43 portals could be blocked in this system. In contrast, in osteoclasts, high expression was expected as gap-junctional CX43 plays a functional role in these cells, being essential in multinucleation and fusion into mature osteoclasts^91^, a process observed in great detail in these constructs (Fig. 2).

Altogether, these results demonstrate a central role of all bone cell types in the early bone signalling events. It was therefore concluded that the most representative organoid conformation was, as predicted, the mixed osteoblast-clast variation, which was used further in development.

### Recreating the physiological and pathological environments

The next stage of development involved providing trabecular organoids with an optimal remodelling-permissive environment by adding missing instructional cues in terms of structure and chemistry, thus leading towards a multi-level system with increased complexity and relevance.

Therefore, mixed osteoblast-osteoclasts constructs were encapsulated into human blood clot-like-fibrin domes reconstituted from human fibrinogen and thrombin, in order to generate proliferation in an endogenous environment that the cells are naturally programmed to respond to. In a parallel experiment, these constructs were embedded for comparison in xenogeneic Matrigel^®^, the current gold standard for organoid proliferation. Because of the mixed phenotypic nature of the constructs and supplementation conditions, the latter were gradually introduced in this system over a period of 10 days, during which they were transferred, as with previous studies^34^ from a spheroid system to a non-adhesive two-dimensional (2D) vessel for 8 days, before being encapsulated into fibrin for a further 8 days. A schematic of the timeline of events is provided in Supplementary Fig. 5.

When imaged at day 19, the morphologies of proliferating masses were observed to be completely different in the anatomical organoids (Fig. 6a) compared to tumorigenic Matrigel^®^ organoids (panel b). Constructs seeded into fibrin develop a significant projection-led expansion into the surrounding matrix (panel a). In contrast, Matrigel^®^ inserted organoids formed packed masses of cells displaying a lobular growth (panel b).

The chemical nature of projections was probed using Micro X-ray fluorescence mapping at day 19 of culture, to allow for an immediate, non-destructive spatial localisation of mineral and matrix components, with different elements coding for distinct anatomical structures (Fig. 6c). As Phosphorus (P) is an element present in both the mineral and cellular component, Potassium (K), one of the most abundant intracellular ions, was complementarily used to spatially distinguish the cells from the matrix and mineral component. While Calcium (Ca) was ubiquitous in this system, we observed a significant phosphorus-led front emerging from trabecular constructs into the fibrin matrix and co-localised with these projections. Some of these deposits were localised in the vicinity of K^+^ containing projections, in areas also rich in Sulphur (S), used as an indication of matrix protein content. Altogether, these results suggested that the projections were involved in a type of mineralised matrix deposition at sites away from the main structure. This was also probed using Micro-Computed Tomography (panel d, e), which allowed the detection of discrete, pocket-like deposits of lower density material forming in lieu of the original fibrin content.

Despite their atypical morphologies, masses of proliferating cells extending into Matrigel^®^ also develop a phosphate and sulphur-rich matrix (Supplementary Fig. 6). Therefore, the Matrigel^®^ model might, nevertheless, be able to simulate an orthotopic tissue environment in vitro, which could recapitulate aspects of the early histotype arising during a cancerous development.

These results also indicate the need for efforts to be directed towards developing organoids using more endogenous matrices that have a better characterised, homogenous biochemistry and clinical applicability, something that has been increasingly recognised and approached^92^ recently.

Unlike blood-derived fibrin, Matrigel^®^ is a highly bioactive, basement membrane derived from the Engelbreth‐Holm‐Swarm mouse sarcoma, containing an abundance of collagens, laminins, glycosylated molecules and basement membrane components^93^. Thus, a large basis for their success in generating relevant organoids is therefore due to this rich template, which molecularly directs an invasive cellular proliferation in a tumorigenic environment. Within the bone organoid field, this is not always desired, especially as the role of certain compounds in vivo are not fully characterised. Certain laminins, for example, produced by osteoblasts, can have a role in supressing osteoclastogenesis in bone^94^.

Fibrin, potentially due to its physiological similarities to those of a real blood clot, has been particularly useful recently in directing embedded cells to form relevant anatomical networks, with recent reports of spherical fibrin beads supporting vascular morphogenesis within^95^, some primitive versions that we were ourselves able to generate using these cells in fibrin in our previous, adapted organotypic bone model^44^.

A further advantage, exploited by other authors as well, is that these fibrin structures can be fused (Fig. 6d) or embedded into further fibrin matrices, making it possible to model complex geometrical structures. From a clinical application perspective, they have also been for many years a delivery vehicle for a wide range of orthopaedic reconstructive procedures^96^ and in this respect, this model represents a preclinical advancement as it comprises a reconstructive bone element, a surgical haemostatic agent, as well as primary gender-matched patient cells, which could be used for personalised and regenerative medicine applications.

### From bone loss to bone remodelling and fracture repair

Following encapsulation in human fibrin, organoids were inserted in the last stage of development into the perfused, analogue microgravity state for 5 days in order to capture the early cellular events induced by unloading, while encapsulated in a tissue-like unit (Fig. 7). Because of this further level of complexity, a second condition was set-up to ensure that any differences recorded (e.g., the potential presence/absence of formations within fibrin) could be accounted for from a gravitational perspective, rather than any hydrodynamic conditions in the reactor. Therefore, constructs were suspended, additionally to the traditional free-fall orbital path along the vertical axis, into a second dynamic condition, revolving within a horizontal plane (Supplementary Fig. 7), which was termed ‘Normal’ Gravity, only to reflect the more traditional distribution of vectors in this system. This parallel system was set-up to ensure that even during short periods of culture, a similar level of perfusion was tested for comparison, in order to check whether any arising differences would be induced by the reactor conditions themselves at this stage. While these effects would not necessarily become evident during shorter time frames, they would need to be a consideration prior to larger, scaled up versions due to the distribution of liquid, as well as before longer cultures.

Following 5 days of orbital trajectory suspension inside the vessels of both conditions, several remarkable features were noticed in constructs. Firstly, areas of dispersed mineral particles resembling bone loss could be observed in the vicinity of constructs and trapped inside the clot-like matrix (Fig. 7a, red boxes). Secondly, significant tubular-like projections can be seen emerging from the mineral interface in organoids (panel a, top and bottom), which, in most cases, extend to the millimetre scale across all axes (panel b). Several additional features were observed in both dynamic conditions. Firstly, masses of mineral can be seen forming from the initial structure, in some cases in a tubular form (panel a, top panel). Secondly, mineral fragments can be seen being transported along these tubules to sites distant from the main structure (panel a, top and bottom). Lastly, large particles of bone mineral, likely originating from the initial structure can be observed trapped within networks formed at the surface of constructs (bottom panel). When these structures were chemically probed at high resolution using Micro-X-Ray Fluorescence, the tubular projections were shown to be composed of protein content co-localised with mineral ions along their lengths (panel c, d). Large spatial maps showed intricate networks of early mineralised matrix structures led by cells and extending from the main organoid structure in both conditions (panel e). To illustrate differences that can be assessed in these constructs, two examples were provided for morphological comparison at an individual construct level in Fig. 7e. These include a construct in the normal gravity condition (top) that contains a higher number of projections compared to a simulated microgravity-cultured construct (bottom), which contains a main tubular structure forming into the matrix, but which displays many possible emerging structures, based on the distribution of potassium (K), in network-like arrangements surrounding the initial structure.

Based on these initial observations on the morphometric characteristics of these tubules forming in constructs, long-term studies could search for differences in the number of projections and their comparative lengths. This could provide further answers regarding the differences in the bone remodelling process between simulated microgravity and the normal context, especially in cytoskeletal organisation, which has been reported to change in microgravity^97,98^.

At the molecular level, a non-quantitative assessment of the protein markers discussed previously, showed the expression of Sclerostin and RANKL in both conditions as noticed previously in multicellular constructs, while in comparison, the expression of the mechanosensing-relevant protein CX43 was less pronounced in both groups, suggesting adaptive changes at different signalling levels (Fig. 7f). Interestingly, the parathyroid hormone receptor protein (PTHR1) was detected in the normal gravity group, however, the specific signal was not categorical in the simulated microgravity group. Further studies could test this expression at different time points to detect any differences in the regulatory pathway controlling the bone remodelling process (either anabolic or catabolic) between the two contexts, especially in relation to sclerostin and RANKL expression.

**Fig. 8:**
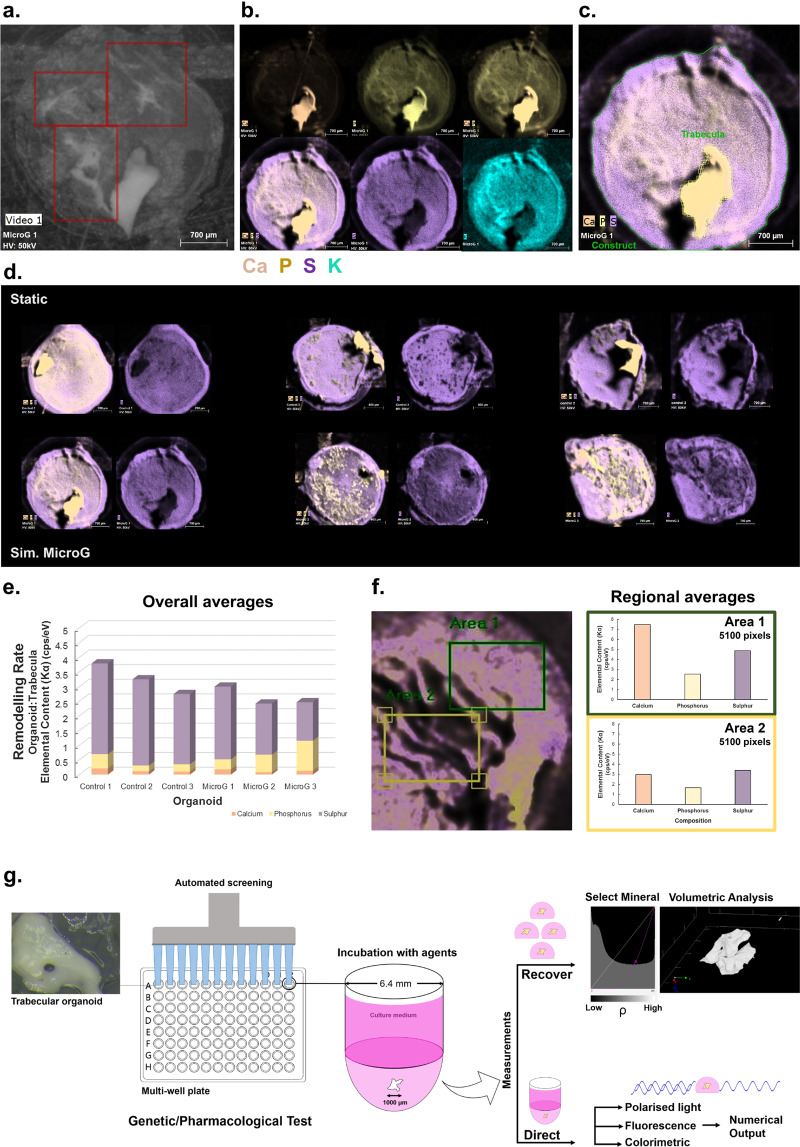
Evolution of constructs and potential applications of the model. **a** Osteoblast constructs that were previously cultured under low-shear conditions for 7 days and subsequently seeded into fibrin matrices, are able to form micro-trabecular shaped soft structures during 3 weeks of culture and to generate nucleation points at sites far away from the initial organoid structure (red squares). This new matrix shows a mixture of protein and inorganic content and a phosphorus-led mineralisation (**b**). **c** It is possible to quantitatively assess the degree of new bone formation by measuring the ratios of elements from the initial construct (yellow shape) compared to the overall organoid (green shape) to assess the degree of change. **d** In these examples from osteoblastic constructs that were either cultured in a static condition (top) or simulated microgravity (bottom) for 7 days and subsequently developed in fibrin for 3 weeks, a rate of remodelling (**e**) could be calculated from the acquired maps by normalising the average elemental content of the organoid to that of the initial trabecular construct. Alternatively, in the example provided in **f** (left) from a previous organoid, regions of interest can be compared within or across samples, provided that the surface area selected is constant (**f**, right). As shown, the differences between Area 1, containing a visibly larger amount of new matrix compared to Area 2, can be successfully translated numerically based on the elemental content. Owing to the small diameter of organoids, constructs can be used with more automated systems to determine the effects of various agents (**g**). Change can be assessed by recovering the organoids and analysing them volumetrically (individually or as a mass) with technologies such as micro-CT, which facilitate a density-based selection (as exemplified here using a function selecting the highest density mineral, i.e., the initial bone). Further tests can be applied, such as illumination with polarised light, which will vary based on the orientation of crystallographic axes within the construct, linked to the degree of new bone formation. Additional colorimetric and fluorescence-based assays specific to bone tissue can be used in conjunction. Scale bars as indicated.

We also determined during pilot work, where constructs were embedded into human fibrin after being exposed to simulated microgravity, that distant spots such as the ones observed in Fig. 7e can advance into more significant structures (Fig. 8a–c) over several weeks, acting as nucleation points for new mineralised matrix formation and generating ‘mini-trabecular’ structures. We have now developed ways to quantify this bone formation process as a ratio of the new mineral formed in the organoid to that of the initial construct, calculated directly from these maps using Micro-XRF (panel c), which will allow more quantitative evaluation of bone formation during long-term cultures or larger scale use. As an example, osteoblastic constructs that were either cultured under static conditions (panel d, top) or simulated microgravity (bottom) for 7 days, followed by culture in fibrin for 3 weeks, were assessed for matrix deposition using this semi-quantitative method. A rate of remodelling can be estimated by normalising the average elemental content of the organoid to that of the initial trabecular construct from the acquired spatial maps for each element. This could demonstrate differences at the matrix-mineral level, where potentially larger deposits are noticeable in the simulated microgravity group (panel d) compared to static, which is translated in the numerical form (panel e). Alternatively, regional comparisons can be conducted within and across samples, provided that the selected region is identical in size (panel f, left and right). This type of numerical extrapolation can also be performed using normal imaging software on an intensity basis, from colorimetric or fluorescence labelled constructs. If available, one of the most rapid and quantitative methods to assess the degree of change in constructs is using micro-computed tomography (which is also described in Fig. 8g), as this technique, which was previously used^44^ to look for mineral deposition in heterogeneous constructs, facilitates the selection and morphometric analysis of specific matrices in composite systems based on their density. For example, the highest density mineral (in this context, the initial trabecula), can be separated from lower density mineral that is newly forming, as well as the lowest density material in the system, which is the initial fibrin scaffold. In this way, the mineral volume (mm^3^) can be obtained and further information on mineral mass could potentially be extracted. Moreover, the same function or function sets can be applied at different time points.

Finally, as the construct diameter matches that of the wells of a 96-slot assay plate, for industrial or large-scale applications, the constructs could be used with more automated (high-throughput) systems in order to test the effects of drugs and other agents during genetic or pharmacological assays (Fig. 8g). Following incubation with the compounds of interest, the change in bone formation can be assessed either by recovering these organoids and analysing them individually or as a mass using Micro-CT as discussed above, or more commonly, directly within the wells through colorimetric or fluorescent detection. Many recent fluorometric detection systems are also able to measure regional intensities within a well, which may provide further levels of information on spatial distribution of new components. Depending on the features available on the automated system, further tests can be applied, such as illumination with polarised light, which could generate different results at different time points depending on the degree of formation of new matrix and therefore the orientation of mineral crystallites as the tissue is maturing.

Overall, this organoid model can provide a window into the early, fine-scale mechanistic events during bone formation. The events recorded in these organoid prototypes mirror both resorption/mineral loss events and the subsequent osteo-proliferation and mineral deposition and therefore, it is likely that constructs recapitulate several steps involved in the bone remodelling process, which can be tuned in the experimental design to simulate both the normal and abnormal physiology.

Nonetheless, the pathological mechanisms in all bone-loss involving conditions, while complex, are ultimately mediated through effects on the bone remodelling cycle and therefore, the restoration of tissue and fractures recapitulates many of the ontological events of embryonic skeletal development. Therefore, the behaviour of cells seen when encapsulating organoids in the blood clot-like fibrin matrices reflect a variety of important previous theories regarding bone remodelling and observations regarding cell behaviours inside haematomas. Indeed, it is now known from mouse models of fracture healing that both events are tightly controlled even at a transcriptional level, as a third of the genes homologous to embryonic stem cells are also involved in fracture healing, that the homeotic genes involved in skeletal development are also induced during repair and that primary morphogenetic pathways, including BMPs, Wnts and FGFs are concurrently expressed during development and fracture repair^99^.

The results also highlight that by using relevant, matched and matured populations inside a native matrix rather than a precursor embryonic-type line (which in this organ model is a necessity due to the mixed origins of effector cells) it can influence more native behaviours and structure formations compared to more developmental models, which can sometimes have a stochastic development and depend largely on self-organisation, that could result in high phenotypic noise^100^.

Secondly, the use of naturally encountered matrices could prevent the pathological transformation of the organoid into a cancerous model as suggested by the Matrigel^®^-encased constructs, thus restructuring the microenvironment, and becoming less representative of the physiological state.

To our knowledge, a similar human cell-based organoid model allowing the observation of detailed morphological and resorption events from bone tissue at the micron scale has not been developed to date, neither a platform that can allow an extensive chemical characterisation of these events. Several osteosphere variations of osteoblastic-osteoclastic constructs have become increasingly available over the years, with or without suspension culture for increased perfusion, and with or without physical barriers of varying biochemistry^65,66,101^, thus simulating individual aspects of pathology and cell interactions. Alternatively, explant chips from rat bones were successfully used with similar bioreactors for understanding some of the effects of weightlessness^102^, however, with the obvious disadvantages of relying on animal tissue, as well as controlling any processing effects on the cells. However, we believe that our results bring the field closer to developing a microphysiological system of bone by providing a simplistic, yet biochemically sufficient remodelling unit, where mature human phenotypes can be tuned and observed in detail. Advancing the design forward from a traditional spheroidal model is essential to allow cells to behave in more native ways and to generate micro-tissues ex vivo that could complement, or replace some of the animal models that currently exist for bone loss research.

## Conclusions

We have presented here results from an early bone organoid prototype that displays promise for studying temporal events in bone remodelling, which may not be accessible using other in vitro and in vivo technologies. In addition, the morphometric features of these constructs make them compatible with a wide range of platforms that can simulate tissue-tissue interfaces and dynamic conditions, including organs-on-chips and thus, could have a major role to play in the preclinical drug development pipeline.

## Methods

### Human cell selection and culture

Human osteoblasts from a female gender, Caucasian donor of an age of 32 years, were acquired from manufacturer Lonza (Lonza Walkersville Inc., USA). The cells were isolated from donated tissue after obtaining legal authorisation for use in research. All culturing reagents were supplied by this manufacturer unless stated otherwise. Cells were cultured in basal medium containing 10% fetal bovine serum (FBS), 0.1% gentamycin sulphate-amphotericin B (GA-1000) and 0.1% ascorbic acid. For differentiation, the medium was further supplemented with hydrocortisone 21 hemisuccinate (200 nM final concentration) and β-glycerophosphate (10 mM final concentration). Hydrocortisone was chosen to induce differentiation as part of a defined supplementation regime (OGM^TM^ SingleQuots^TM^) from the manufacturer Lonza (Lonza Walkersville Inc., USA), which tested and certified its use with the primary human osteoblastic cells as leading to alkaline phosphatase positive (immunofluorescence) and mineralisation positive (von Kossa staining) cells. Therefore, the Hydrocortisone-β-Glycerophosphate regime was employed both to ensure the expected differentiation and mineralisation, as well as to minimise potential variability due to using a differentiation diet containing an alternative steroid, e.g., dexamethasone, which is commonly used. Cells were cultured and used at passages 3–4 to develop organoids.

A population of human osteoclast precursors from a female donor were also acquired from manufacturer Lonza (Lonza Walkersville Inc., USA), which were isolated following acquisition of legal authorisation for use in research. These cells were certified as TRAP-competent using staining and guaranteed as ≥40% or 100 TRAP positive, as well as ≥100,000 RFU Osteoclast Precursors Differentiated cells using an OsteoLyse Assay test (Lonza Walkersville Inc., USA). Cells were cultured in basal medium containing 10% FBS, 1% Penicillin-Streptomycin, 1% l-Glutamine, 0.1% rhM-CSF and 2 µg of soluble RANK Ligand. These cells were used directly following resuspension in culture medium to remove the cryopreservation agent or following one differentiation cycle, when they were lifted using standard subculturing protocols.

### Human trabecular organoid prototype development

Trabecular fragments (500–1000 µm) used as grafting materials for human maxillofacial reconstruction (Cerabone^®^, Botiss Biomaterials, GmbH, Germany), originating from the femoral heads of xenogeneic sources (New Zealand cattle) were used as the base for organoid generation. These fragments are thermally heated up to >1200 °C to remove the organic component according to ISO standards DIN EN ISO 22442-1, DIN EN ISO 22442-2 and DIN EN ISO 22442-3, leaving solely a phase of hydroxyapatite mineral in their structure.

Cells were seeded on the surface of trabeculae using an inverted drop culture, where 10 µl drops containing the trabecular structure and a cell suspension were incubated in an inversed manner for a period of 48 h to direct attachment using the gravitational pull and maximise colonisation. The drops were deposited on the inside surface of a tissue-culture treated 60 mm petri dish, while 5 ml of phosphate buffered saline were added to the bottom of the dish to create a humid environment/a hydration chamber, thus preventing evaporation. Individual osteoblastic, pre-osteoclastic and mixed populations were cultured on these scaffolds. Mixed cells organoids were generated using a ratio of 1:2-1:3.5 osteoclast:osteoblast. Cell concentrations were adjusted prior to mixing and were applied as 5 µl of each population to generate a 10 µl drop. In encapsulated forms, 2500 osteoclast precursors to 5000 osteoblasts were used (1:2), while in non-encapsulated forms 500:1250 (1:2.5) and 350:1250 (1:3.5) were used. The initial optimisation work using non-encapsulated constructs started with a lower proportion of osteoclasts to osteoblasts to determine a minimum amount of cells that can elicit an effect and to observe the colonisation of the mineral surface. This number was subsequently increased to 1:2 osteoclasts: osteoblasts in encapsulated form.

### Encapsulation of organoids

Mixed-cell organoids were individually transferred to the wells of 48-well non-adherent culture plates (Greiner Bio-One, Austria) and their medium was replaced every other day. Organoids were handled by aspirating using an 1000 µl pipettor connected to tips that had ~1 cm of the tip removed with a sterile scalpel. Following 8 days of acclimatisation into an osteogenic environment containing 200 nM Hydrocortisone and 10 mM β-Glycerophosphate in a low-binding environment (following 10 days of total culture), constructs were transferred into human blood clot-like fibrin domes, generated using a previously described technique for producing bovine fibrin^44,46^. Essentially, the scaffold is created using the reaction between the (in this case human-derived) endolytic serine protease thrombin, which selectively cleaves the Arg-Gly bonds of fibrinogen to form fibrin. High activity thrombin from human plasma (≥1000 NIH units/mg protein) (Sigma Aldrich–Merck KGaA, Darmstadt, Germany) was reconstituted using 0.1% albumin from human serum (Sigma Aldrich–Merck KGaA, Darmstadt, Germany) in 5 ml F12K Nutrient Mixture with Kaighn’s Modification (1X) (Gibco Life Technologies, Paisley, United Kingdom) to achieve a final concentration of 200 units per ml. Fibrinogen from human plasma (50–70% protein, ≥80% of protein clottable) (Sigma Aldrich–Merck KGaA, Darmstadt, Germany) was reconstituted in F12K Nutrient Mixture (1X) with Kaighn’s Modification (Gibco Life Technologies) at a ratio of 20 mg/ml. Thrombin was added to a solution containing the cell culture medium at a ratio of 50 μl/ml. The anti-fibrinolytic agents aminohexanoic acid (200 mM) (Sigma Aldrich-Merck KGaA, Darmstadt, Germany) and aprotinin from bovine lung (10 mg/ml) (Roche Diagnostics GmbH, Mannheim, Germany) were added to the thrombin solution at a ratio of 2 μl/ml in order to reduce the degradation rate of the fibrin gel. Each scaffold was generated by mixing a ratio of 2.5 thrombin solution:fibrinogen as per previous methods to generate an 100 μl construct (71.43 μl thrombin solution to 28.57 μl fibrinogen). Gels were allowed to polymerise for 30 min at 37 °C inside 96-well U-bottom tissue-culture plates, generating dome-shaped structures. Constructs were pipetted to the surface of polymerised clots and 100 µl of medium was provided to each construct. Subsequently, the constructs became transiently encapsulated into the structure over the following day, which is allowed by the self-healing property of this versatile polymer and were cultured for a total of 8 days.

In parallel, an experiment was set-up for comparison where organoids from the same population were transferred following inversed drop culture into Matrigel^®^ domes. These were created using similar methods described previously^34^, by pipetting into 30 µl of cold Matrigel^®^ droplets on a sheet of Parafilm with small 5 mm dimeter dimples, formed by pressing with a standard sterile rayon swab (155C) (Copan Diagnostics Inc., California, USA) against the indentations of a 10 µl pipette tip rack (Starlab, United Kingdom). These droplets were allowed to polymerise at 37 °C and were subsequently removed from Parafilm and transferred into osteoblastic-osteoclastic medium and fed every other day.

### Simulation of mechanical unloading conditions

In order to expose organoids to a reduced mechanical environment, a rotating bioreactor (NASA/Synthecon Inc. Houston, Texas) was used to subject trabecular organoids, in a direct of encapsulated form, to a state of orbital buoyancy, experiencing minimal shear and cancelling the gravitational pull. Constructs were inserted into single-use 50 ml High Aspect Ratio Vessels (HARVs) containing a large silicone membrane allowing for enhanced gas exchange. These chambers were filled with culture media by simultaneously operating two 10 ml Luer Lock syringes connected to the vessel valves, out of which one was used to inject the culture medium and the second to capture the air pockets until they were completely removed. Constructs were pipetted into these chambers using the vessel’s main port, which was sealed prior to attachment to the rotary engine. Vessels containing the encapsulated constructs and anchorless constructs were rotated at 23 RPM constant. The vessels containing non-encapsulated constructs were rotated at 29 RPM constant to achieve orbital buoyancy and prevent sedimentation. To determine the ideal rotational speed for the culture vessel, the dynamic behaviour of constructs was observed as the speed was gradually increased using the tachometer on the control unit, from 0 RPM, until the constructs achieved terminal velocity in uniform, circular, free-falling trajectories. Rotational speeds below and higher than the ideal value lead to similar, though inconsistent trajectories.

In non-encapsulated construct work, comparisons were performed between the simulated microgravity condition and the equivalent, static controls. In encapsulated constructs, due to the presence of the fibrin matrix, a second dynamic condition was used in parallel to simulated microgravity, where a second reactor was run horizontally and in parallel to ensure that any potential differences obtained could be related to the gravitational context rather than the conditions inside the reactor vessel.

### Imaging of whole organoids

Cells in fixed constructs were fluorescently labelled with the phallotoxin Phalloidin conjugated to Alexa Flor 555 for cytoskeletal labelling (Thermo Fisher Scientific, Massachusetts, USA), diluted to a concentration of 200 units per ml (6.6 μM) and 3U were applied to each sample. The dye was used in conjunction with the nuclear/DNA staining dye 4′,6-Diamidine-2′-phenylindole (DAPI) (Thermo Fisher Scientific, Massachusetts, USA).

Brightfield images of live cells and organoids were captured using an EVOS XL Core Imaging system (Invitrogen, Life Technologies, Oregon, USA).

Calcein acetoxymethyl (AM) was used to fluorescently label live cells at a ratio of 1–2 μl/ml and was allowed to incubate with organoids for 20 min before imaging. Cellular nuclei in organoids were imaged using NucBlue^TM^ Live Cell stain containing Hoechst 33342 (2’-[4-ethoxyphenyl]-5-[4-methyl-1-piperazinyl]-2,5’-bi-1H-benzimidazole) (Invitrogen, Life Technologies, Oregon, USA). The reagent was added at a ratio of 2 drops/ml and incubated with the cells for 15 minutes before imaging as a single label or multiplexed with additional dyes. Cellular structures in organoids (tubulin, actin) were imaged using commercial dyes CellLight^®^ BacMam 2.0 (Thermo Fisher Scientific, Massachusetts, USA), containing fusion constructs of the plasma membrane myristolyation/palmitoylation sequence from human Lck tyrosine kinase (0.9 kD, *H. sapiens*) and TagRFP; and of N-terminus of β-tubulin (49.8 kD, *H. sapiens*) and emGP. The reagents were incubated with the cells at a ratio of 50 particles per cell (PPC) for 18 h at 37 °C to allow cells to express RFP and GFP and were then imaged using an Olympus Fluoview FV1000 confocal laser scanning microscope (Olympus, Tokyo, Japan) equipped with a multi-line argon laser FV5-LAMAR/LAMAR-2 and a Helium-Neon Green Laser FV5-LAHEG-2/FV5-LAHEG. Images acquired from excitation at 405/488/543 nm wavelengths were collected in individual channels and combined using the Fluoview FV10-ASW software, version 4.2 (Olympus, Tokyo, Japan).

Wide-field fluorescence images of encapsulated live organoids were acquired using an EVOS fluorescence cell imaging system (M5000) (Invitrogen, Life Technologies, Oregon, USA), capturing transmitted light and two fluorescence channels (DAPI and GFP).

### Chemical mapping using micro X-ray fluorescence spectroscopy (micro-XRF)

A micro X-ray fluorescence (μ-XRF) system (M4 Tornado, Bruker Instruments, Germany) was used to generate spatially resolved chemical maps of organoids, scaffolds and bone tissue and to investigate the formation of matrix and cellular structures within fibrin matrices by coding for CAlcium, Phosphorus, Sulphur and Potassium, present in these structures. The machine contains a rhodium μ-focus X-Ray tube and a polycapillary lens, used to focus the X-Rays to a spot size of 25 μm. Recordings were taken without prior sample processing, at room temperature and under vacuum conditions. The X-Ray tube operated at a voltage of 50 kV and 200/400 μA anode current. High-resolution maps were acquired using a spot size between 4–20 μm and exposure times of 2–50 ms/pixel. The same settings were used for all constructs in the same group of investigation. Elemental maps were formed in real time by integrating the photon counts around the emission lines of Ca (Kα), P (Kα), S (Kα), K (Kα), generating an image where pixel intensity was proportional to the number of X-Ray counts/s per electronvolt (eV) from each measured point on the construct. Thus, pixel intensity increased with X-Ray counts, with maximum pixel intensity normalised to the highest count rate per eV for each element of interest, across the entire construct.

### Microtomographies of constructs

A micro-computed tomography system (μCT) (SkyScan 1172, Bruker Instruments, Germany), was used to evaluate mineralisation and matrix formation in encapsulated organoids on a density basis. Constructs were removed from culture medium and fixed using 4% paraformaldehyde for 1 h, thoroughly rinsed using PBS and scanned as a fused mass by placing vertically inside a 7 ml polystyrene Bijou container (Thermo Fisher Scientific, Massachusetts, USA) on the rotating stage inside the scanner (positions: 91.3 mm object to source and 217.11 mm camera to source). High-resolution scans were performed at ambient pressure using the cone-beam imaging system, composed of a Hamamatsu X-Ray source with a voltage of 80 kV and a tube current of 100 μA. The X-Ray detector consisted of an 11 Mp X-Ray camera of a 9.01 μm pixel size, generating images of 3.79 μm pixel size. Two-dimensional cross-section slices of the constructs were acquired using a rotation step of 0.1 degrees, with two frames averaging per step and an exposure time of 1180 ms. Acquired images were three-dimensional (3D) reconstructed using the Bruker micro-CT NRecon Software (v. 1.7.1.0). For removal of scanning artefacts, the beam hardening correction was set to a value of 30% to correct for the surface-to-depth density gradient caused by increased X-Ray attenuation at the surface of the bone constructs. A ring artefact correction was set to a level of 31. The smoothing parameter was adjusted to a value of 4. Trabeculae without cells were scanned as a mass and reconstructed using the settings described above, at 0.2 degree rotation (44.02 mm object to source distance), and an image pixel size of 1.83 μm. Transfer functions were created in the CTVox software that allowed segmentation of the high-density matrix components, as well as for creating colour-coded versions of the multiple components in constructs.

### Surface characterisation using scanning electron microscopy (SEM)

Constructs were removed from culture and dried overnight at room temperature, as preserving cells was not required for surface analysis. They were then mounted on top of double adhesive carbon discs attached to aluminium stages. The structures were sputter coated with either Ag or Pd and secondary electron micrographs of the surface were acquired under vacuum, with a beam power of 15 kV using a Hitachi TM3030Plus tabletop SEM microscope (Hitachi, Tokyo, Japan). A selection of constructs from these populations were used for mechanical testing at the end to ensure correspondence between the resorption and mechanical datasets.

### Mechanical testing of organoids

A Zwick/Roell Z030 universal mechanical tester was used to compare compressive force resistance across different construct groups. Compression was conducted using a 50 kN load cell on a 30 kN frame. Organoids were placed on the bottom plate while the top plate was accelerated at a speed of 2 mm/min and compressed the sample until terminal failure of the structure was achieved.

### X-ray diffraction analysis

X-ray diffraction analysis (XRD) was performed on the trabecular fragments using a D8 Advance diffractometer instrument equipped with a Cu X-ray source (1.5418 Å) and LYNXEYE (1D mode) detector (Bruker Instruments, Germany). Trabeculae were analysed intact and diffraction patterns were acquired between 10–50° 2*θ*, with a step size of 0.02° and a step time of 0.3 s. The diffraction pattern was matched against that of biological apatite using PDF No. 00-046-0905 from the integrated database from International Centre for Diffraction data.

### Sample processing for molecular characterisation

To analyse the secretome of constructs exposed to simulated microgravity conditions, medium from their culture environment was concentrated, due to the large volume of liquid that they were suspended in inside the rotating reactor vessels. 20 ml of incubation medium (40% of the total volume that each group of constructs was cultured in) was acquired and centrifuged at 220 G for 10 min to remove large particles such as cell membranes. The pellets were discarded and 15 ml of the supernatant were placed inside the upper chamber of individual Pierce™ Protein Concentrator PES 10,000 MWCO tubes (Thermo Fisher Scientific, CA, USA) to concentrate the proteins within the molecular size range of interest. The samples were concentrated to 0.5 ml and were resuspended in 10 ml PBS and used for immunopurification.

For western blotting analysis, the medium was centrifuged at 220 × *g* for 10 min and pellets were snap frozen at −80 °C for 30 min. They were then rapidly pulverised using a multi-sample biopulverizer (BioSpec Products Inc., OK, USA), suspended in 1.4 ml PBS and frozen at −20 °C until processing.

### Western blotting analysis

All samples were pre-labelled with a total protein stain, Cyanine 5, for 30 min (detected at 700 nm). Following labelling, samples were incubated with dithiothreitol (DTT) and lithium dodecyl sulphate (LDS) loading buffer and boiled for 5 min at 90 °C. Samples were then spun at 14,000 x *g* for 1 min prior to loading onto 4–12% Bis-Tris 12 well NuPAGE gels run in MOPS (3-(N-Morpholino)propanesulfonic acid) buffer, set at 180 V for 55 min. Twenty microlitres of the protein suspension was loaded per well from samples and 0.1 μg of pure protein. Gels were transferred onto nitrocellulose at 100 V for 1 h and blocked with 5% BSA for 2 h. Primary antibodies diluted as 1:1000 in PBS + 0.1% Tween were incubated with the blots for 1 h. The primary antibodies used were polyclonal IgG rabbit anti-human PTH1R (N-terminus of human parathyroid hormone receptor type 1); anti-human SOST (sequence within the centre region of human Sclerostin) (Thermo Fisher Scientific, Massachusetts, USA); anti-CX43 (phosphopeptide corresponding to amino acid residues surrounding the phosphoserine 368 of connexin 43) (Invitrogen, Life Technologies, Oregon, USA); and monoclonal IgG mouse anti-human RANKL (clone 6A12.1, targeting an epitope within 95 amino acids from the C-terminal region) (Merck KGaA, Darmstadt, Germany). Fluorescent secondary antibodies—donkey anti-rabbit or anti-mouse IgG (800 nm)—were incubated as 1:10,000 in PBS + 0.1% Tween for 1 h. Rigorous washing with PBS + 0.1% Tween was carried out between steps. Blots were imaged on a LI-COR Odyssey CLx (Licor Biosciences, Nebraska, USA) gel scanner at 700 nm (for total protein) and 800 nm (for specific antibody signal). A Chameleon duo pre-stained protein ladder (Licor Biosciences, Nebraska, USA) was used for reference. All blots derived from the same experiment were processed in parallel.

### Immunopurification of target proteins

Proteins of interest (Sclerostin, RANKL) were isolated from the concentrated culture medium of constructs by immunoprecipitation, using methods described previously^45^, using 2.8 μm superparamagnetic Dynabeads (Invitrogen, Thermo Fisher Scientific, CA, USA), covalently coupled to Protein G on their surface (~17 kDa). Goat polyclonal IgG antibodies against human sclerostin (peptide with sequence C-PRARSAKANQAELEN, from the C-terminus of SOST) and mouse IgG anti-RANKL (as above) were used to isolate the proteins. Antibodies were attached to the magnetic beads through their Fc region during a 15 min incubation with rotation at room temperature, by diluting into 200 μl of PBS containing 0.01% Tween-20 and 0.09% sodium azide, in which 1.5 mg magnetic beads were resuspended (Invitrogen, Thermo Fisher Scientific, CA, USA). The complex formed was washed by resuspending in 200 μl buffer and 1000 μl of concentrated medium was added to the formed Dynabeads-Ab complex. Samples and complex were incubated for 15 min at room temperature, with rotation. The beads-Ab-protein complex was subsequently washed four times using PBS washing buffer (Invitrogen, Thermo Fisher Scientific, CA, USA). Finally, the Ab-protein complex was eluted from the beads though the addition of 20 μl elution buffer (Invitrogen, Thermo Fisher Scientific, CA, USA). For gel electrophoresis, samples were resuspended in a loading mixture containing 2.5 μl NuPAGE LDS Sample buffer (4X), 1 μl NuPAGE Reducing Agent (10X) (all from Thermo Fisher Scientific, CA, USA) and 6.5 μl dH_2_O and were incubated for 10 min at 70 °C. Samples were loaded onto NuPAGE Novex 4–12% Bis-Tris Protein and were run at 160 V for 60 min using MOPS buffer. Five-hunded microlitres NuPage Antioxidant (Thermo Fisher Scientific, CA, USA) was added to the buffer to maintain the reduced state of proteins during protein gel electrophoresis. A Novex Sharp pre-stained protein standard was used for reference (Thermo Fisher Scientific, CA, USA). Pure medium from each category was run in parallel to isolated protein samples.

### Statistical analysis

Force measurements were acquired in triplicate (from three constructs). All data is presented as mean ± SD. Statistical analysis was performed using a one-tailed distribution *t*-test, with a homoscedastic variance assumed. A *p*-value lower than 0.05 was chosen for determining significance (MS Excel, Washington, USA).

### Reporting summary

Further information on research design is available in the Nature Research Reporting Summary linked to this article.

## Supplementary information

Supplementary Information

Reporting Summary

## Data Availability

The data supporting the findings of this study is available within the paper and its supplementary information files.
