# Supplementary material for: Trabecular bone organoids: a micron-scale ‘humanised’ prototype designed to study the effects of microgravity and degeneration
Source: NPJ Microgravity. 2021 May 21;7:17. doi: 10.1038/s41526-021-00146-8 (PMC8140135; doi:10.1038/s41526-021-00146-8)
Supplement: Supplementary file 1 — Supplementary Information [file 41526_2021_146_MOESM1_ESM.pdf]

Supplementary information

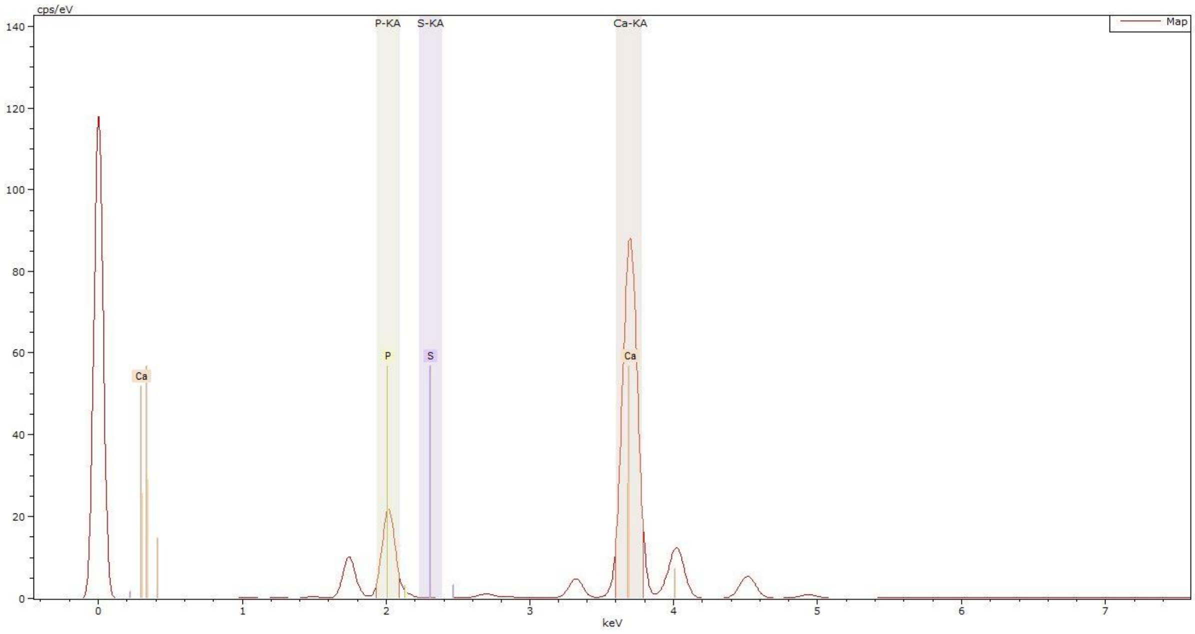

Supplementary Figure 1. Elemental composition of micro-trabeculae. Overall Micro-X-Ray Fluorescence spectrum of a trabecular particle analysed, indicating large amounts of Calcium (**beige**) and Phosphorus (**yellow**), as well as minimal traces of the element Sulphur (**purple**).

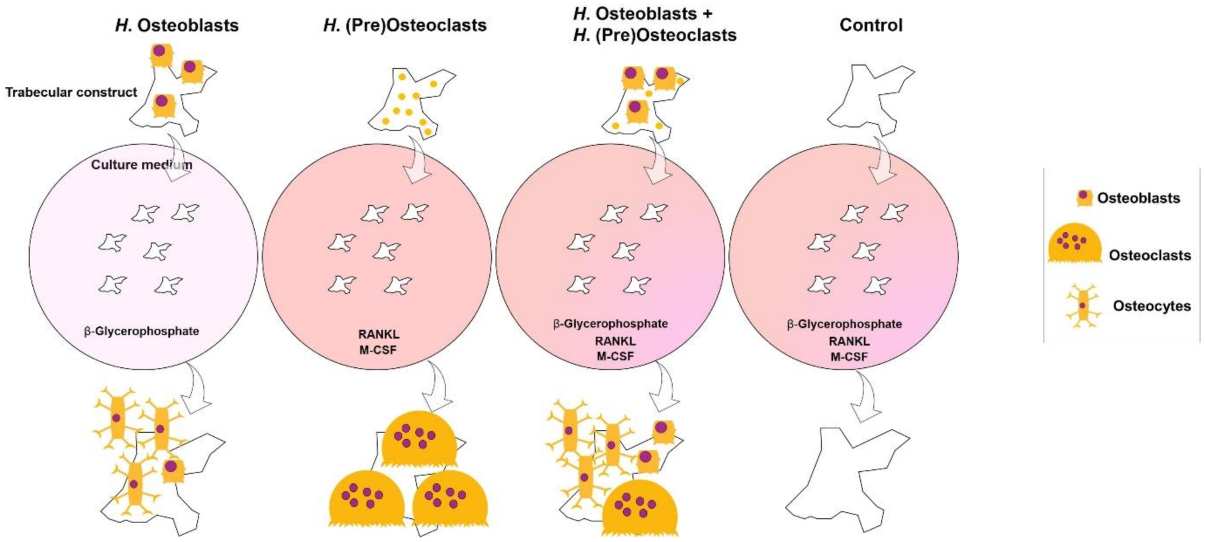

Supplementary Figure 2. Schematic of the organoid set-up conditions and supplementation regime used to culture cell-seeded trabecular constructs. Human gender-matched (female) osteoblasts and mature/pre-osteoclasts are seeded onto the micro-trabeculae as individual or mixed populations. Mixed constructs are designed to ultimately contain all three types of effector cells encountered in bone. The culture medium and pro-differentiation supplements in mixed constructs are added proportionally to the ratio of cells used, to generate populations of mature osteoclasts (using RANKL and M-CSF), osteoblasts and osteocytic cells (using an osteogenic mixture including β-Glycerophosphate). Controls are grown under the same conditions as mixed cell-constructs, without the addition of cells.

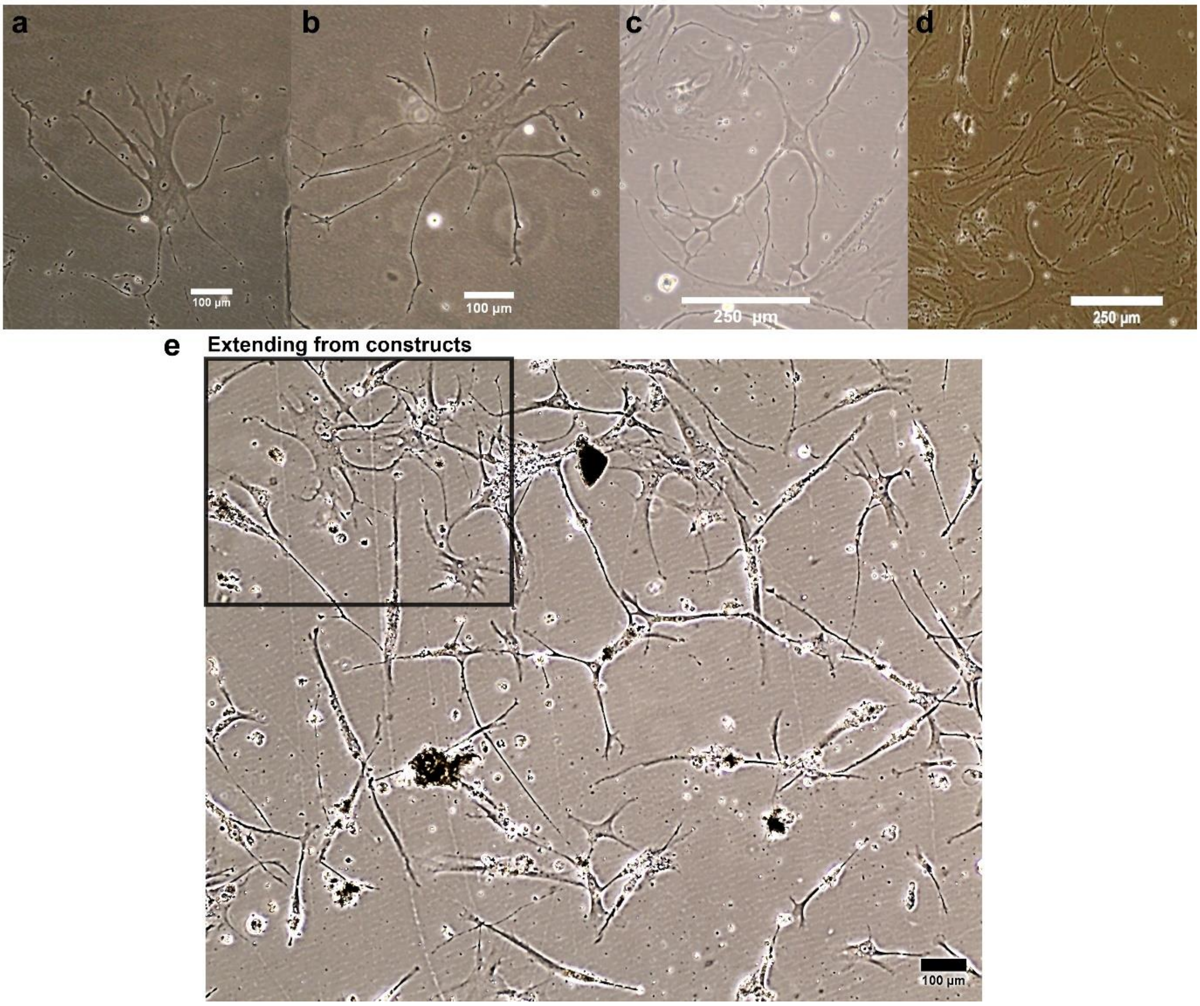

Supplementary Figure 3. Morphologies of human osteoblastic cells cultured *in vitro*. The morphologies of cells cultured in osteogenic conditions become highly-dendritic and neuronal (**a**-**d**), indicating a transition from a stromal phenotype into a pre-osteocytic and osteocytic phenotypes. The cells cultured in three-dimensional trabecular constructs and placed in standard culture dishes can proliferate and give rise to networks and typical mature osteocytic morphologies at distant sites (**e**, **black box**). Scale bars as indicated.

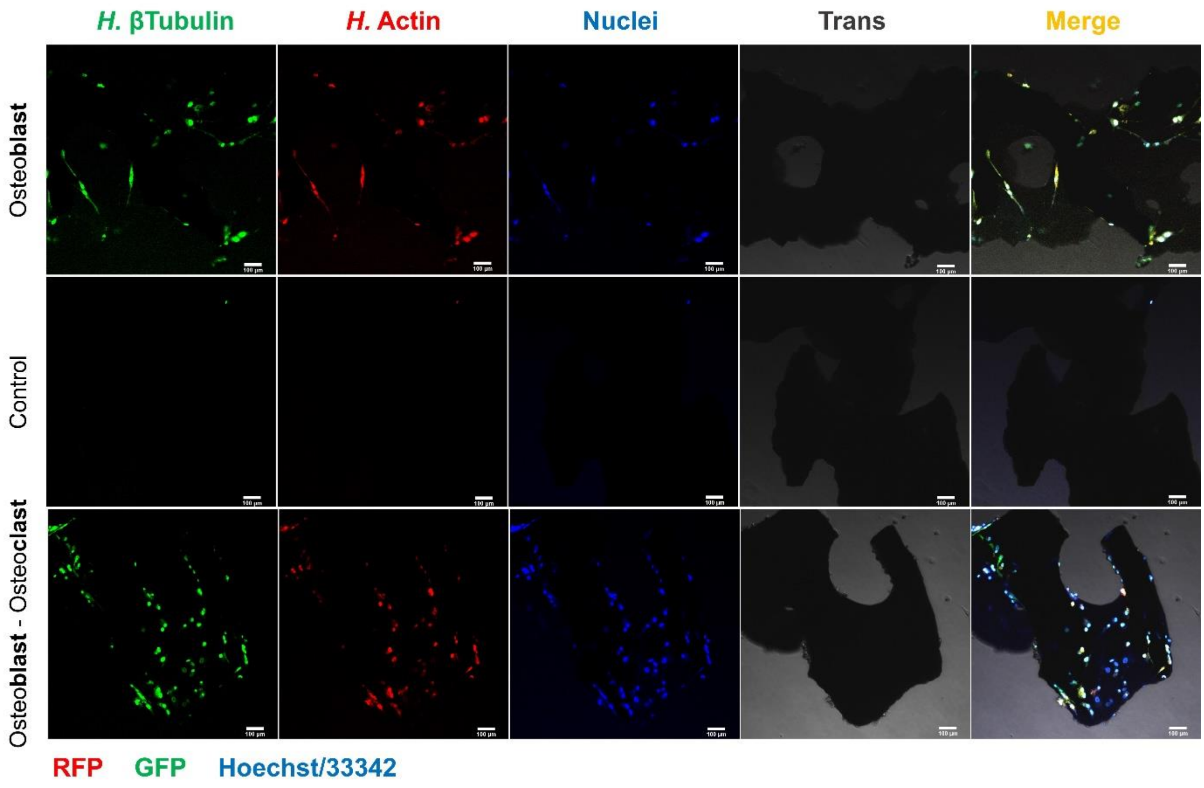

Supplementary Figure 4. Morphologies of cells in organoids following 8 days of culture. A mixture of cell morphologies can be achieved in constructs (**top, bottom rows**), with some mesenchymal stromal morphologies still present on the surface of osteoblastic constructs (**top**) indicative of a mixed osteoblastic-osteocytic population. In addition, mixed osteoblast-osteoclast constructs (**bottom**) display further morphologies including small round cells, indicative of the maturing monocyte population. Scale bars as indicated.

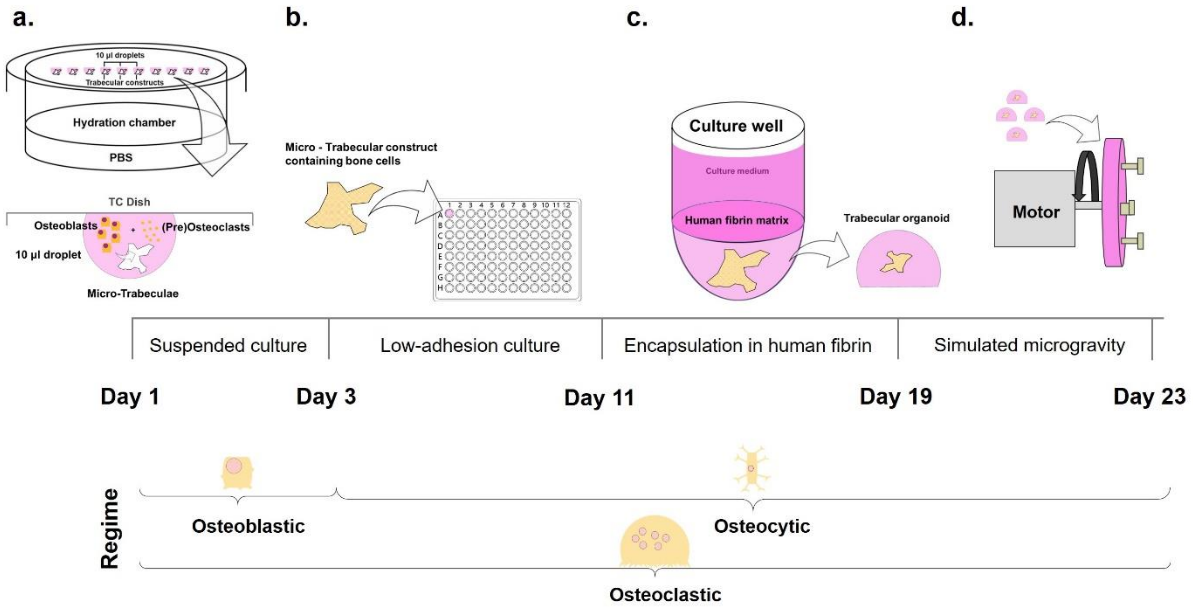

Supplementary Figure 5. Encapsulation of trabecular organoids and timeline of events. Mixed osteoblastic-osteoclastic organoids are transferred following two days of self-assembly inside an inversed drop culture system, into a non-adherent tissue culture dish, where they can gradually adapt from a partially osteoblastic to a pro-osteocytic supplementation regime. Following 8 days of culture in these conditions, they are transferred into human fibrin domes, where they are allowed to accommodate, proliferate and develop networks. Following 8 days of culture in this matrix, they are suspended for 5 days inside a NASA/Synthecon reactor, to investigate the early cell response to unloading. A full osteoclastic regime is provided throughout the entire culture to ensure the correct molecular environment of the monocyte population.

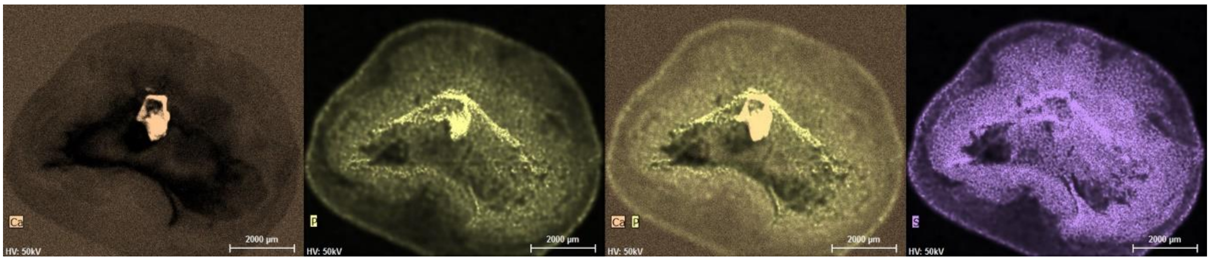

Supplementary Figure 6. Mineral development in tumorigenic Matrigel®-encapsulated organoids. Constructs cultured in a Matrigel® matrix display a progressive expansion of mineralised matrix from the trabecular surface into the surrounding soft scaffold at day 15 of culture. These regions are also rich in sulphated content, indicative of protein deposition. Scale bars as indicated. The map was acquired using Micro-XRF, at 20 μm pixel size and 10 ms per pixel exposure time, 50 kV and 400 μA, under vacuum.

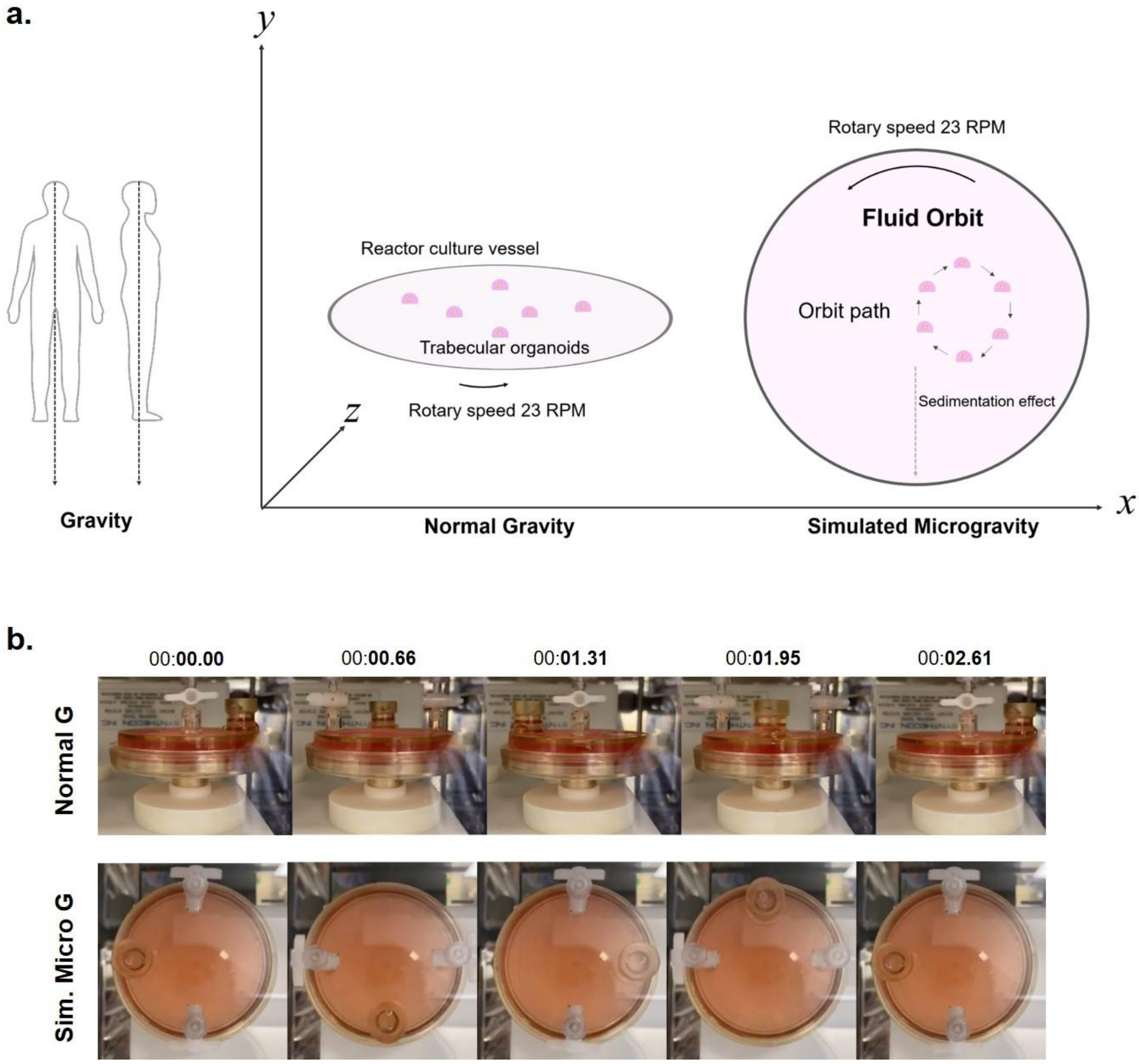

Supplementary Figure 7. Design of a disuse-simulating environment. In order to simulate a reduction in the forces experienced by trabecular bone, which is heavily adapted to withstand the gravitational force (**a**), encapsulated organoids were placed in a NASA/Synthecon Bioreactor, operating on two axes – a traditional, vertical orientation, allowing the organoids to form an orbital buoyant trajectory, and a horizontal orientation (termed 'Normal' Gravity, as the constructs experience the gravitational pull in a more conventional way) to control for any arising differences due to the perfused vessel itself. During revolution at 23 RPM (**b**), constructs are suspended (Sim. Micro G) or mixed in the culture medium.

a. Cell membrane protein extracts

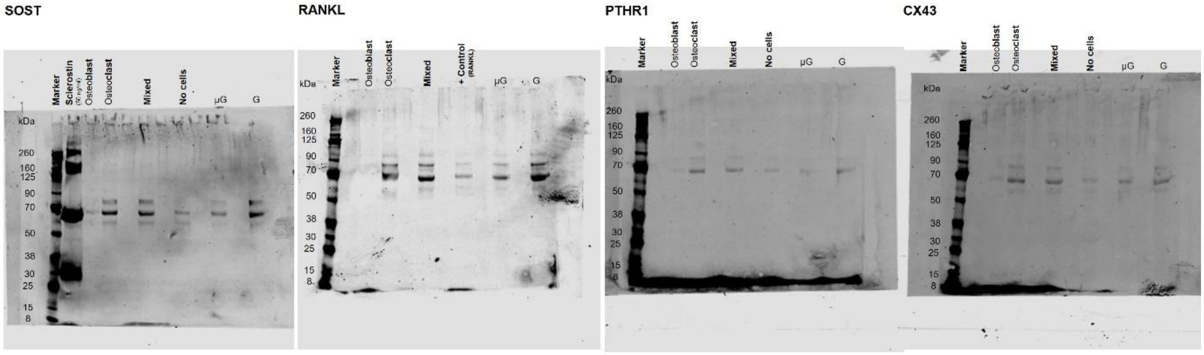

b. Immuno-purified from medium

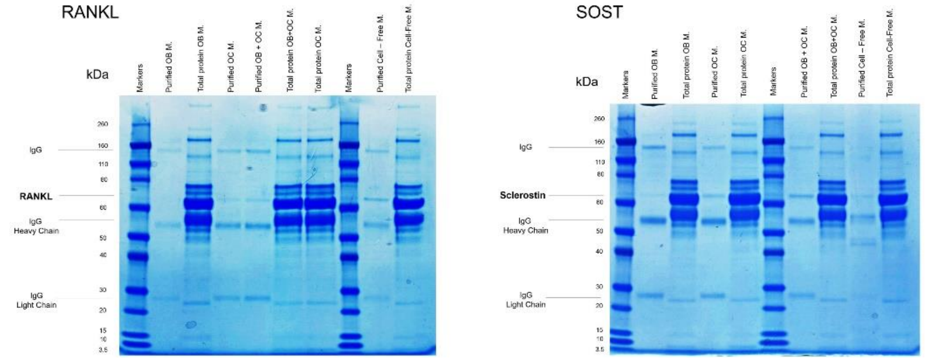

Supplementary Figure 8. Western Blots (**a**) and protein electrophoresis gels (**b**) for cell membrane protein extracts probed for Sclerostin, RANK ligand, Parathyroid hormone receptor 1 and Connexin 43 (**a**) and Sclerostin and RANK ligand in immuno-purified complexes (**b**). Samples were collected from the culture of osteoblastic, osteoclastic, mixed-cell and control constructs grown in simulated microgravity conditions (first study); and mixed-cell encapsulated constructs cultured in simulated microgravity (μG) and normal gravity (G) (second study).
